# Cadherins, Selectins, and Integrins in CAM-DR in Leukemia

**DOI:** 10.3389/fonc.2020.592733

**Published:** 2020-12-10

**Authors:** Hye Na Kim, Yongsheng Ruan, Heather Ogana, Yong-Mi Kim

**Affiliations:** ^1^Children’s Hospital Los Angeles, Keck School of Medicine of University of Southern California, Cancer and Blood Disease Institute, Los Angeles, CA, United States; ^2^Department of Pediatrics, Nanfang Hospital, Southern Medical University, Guangzhou, China

**Keywords:** leukemia, cell adhesion molecules, cell adhesion-mediated drug resistance, chemoresistance, bone marrow microenvironment

## Abstract

The interaction between leukemia cells and the bone microenvironment is known to provide drug resistance in leukemia cells. This phenomenon, called cell adhesion-mediated drug resistance (CAM-DR), has been demonstrated in many subsets of leukemia including B- and T-acute lymphoblastic leukemia (B- and T-ALL) and acute myeloid leukemia (AML). Cell adhesion molecules (CAMs) are surface molecules that allow cell–cell or cell–extracellular matrix (ECM) adhesion. CAMs not only recognize ligands for binding but also initiate the intracellular signaling pathways that are associated with cell proliferation, survival, and drug resistance upon binding to their ligands. Cadherins, selectins, and integrins are well-known cell adhesion molecules that allow binding to neighboring cells, ECM proteins, and soluble factors. The expression of cadherin, selectin, and integrin correlates with the increased drug resistance of leukemia cells. This paper will review the role of cadherins, selectins, and integrins in CAM-DR and the results of clinical trials targeting these molecules.

## Introduction

Despite the improved overall survival of leukemia patients, relapsed and refractory leukemia still remain a problem. Chemoresistant minimal residual disease (MRD) contributes to the recurrence of the disease. Patients with relapsed leukemia in the bone marrow (BM) have worse outcomes than patients with relapses in the central nervous system or testis (1), suggesting the contribution of the BM to the progression and aggressiveness of the disease. Indeed, the BM microenvironment is known to govern leukemia quiescence (2–4), proliferation (5), drug resistance (6), and leukemogenesis (7). Leukemia cells communicate with BM through surface molecules called cell adhesion molecules (CAMs). The CAM-mediated interaction of leukemia cells with the surrounding microenvironment contributes to drug resistance, which is called cell adhesion-mediated drug resistance (CAM-DR). Surface molecule overexpression and CAM-DR have been addressed in many subtypes of leukemia, including B- and T-acute lymphoblastic leukemia (B- and T-ALL) and acute myeloid leukemia (AML). CAM-DR is one mechanism through which leukemia cells obtain chemoresistance, and resistant clones will result in the recurrence of the disease. Due to aberrant expression, CAMs serve not only as a prognostic tool for detecting MRD in leukemia but can also be targeted to sensitize drug-resistant cells to chemotherapy (8–11). CAM inhibition in leukemia is being actively evaluated in preclinical and clinical studies. In this review, we will focus on the major groups of CAMs—cadherin, selectin, and integrin—and their role in drug resistance in leukemia.

## Leukemia And Leukemia Stem Cells

Leukemia is a type of Cancers that affects a patient’s blood and bone marrow. Leukemia can be categorized into different subtypes depending on the progression of the disease (acute and chronic) or a lineage and developmental stage of cells (myeloid or lymphoblastic). Four main subtypes of leukemia that will be discussed in this review are: acute lymphoblastic leukemia (ALL) (12–14), AML (15, 16), chronic lymphocytic leukemia (CLL) (17, 18), and chronic myeloid leukemia (CML) (19, 20). Leukemic cells have been described to interact with and remodel BM to support leukemic cell expansion and survival (21).

LSCs are capable of self-renewal and thus able to maintain survival in optimized *in vitro* co-culture systems and in immunocompromised mice, which have been defined in AML and CML (22). CAMs play an important role in the interaction between LSCs and the hematopoietic niche (23, 24). Firstly, it has been shown that N-cadherin positive CD34^+^ CD38^−^ LSCs population has a critical role in the development of AML (25, 26). Moreover, the downregulation of E-cadherin suppressed the adhesion of AML cells to BM-derived MSCs and enhanced the anti-leukemic effect of cytarabine (27). Secondly, in an AML mouse model, LSCs adhered to the vascular niche which protected LSCs from chemotherapy through E-selectin/E-selectin ligands and this effect was ameliorated by GMI-1271 (28). Thirdly, VLA-4 is one of the most prominent integrins involved in LSCs (24). Recently a study showed that inhibition of Kindlin-3-mediated VLA-4 adhesion mobilized LSCs in the BM and prolonged survival of mice with CML (29). Furthermore, the integrin αVβ3 was expressed in particular on CD34^+^ cells in AML with NPM1 mutation (30).

## Cell Adhesion Molecules In Leukemia

BM is a complex tissue with various components. Mesenchymal stromal cells (MSC) (31, 32), endothelial cells (4, 33), osteoblasts (34), adipocytes (35), neurons (36), Schwann cells (37), and megakaryocytes (38) comprise the endosteal and vascular BM niches. Soluble factors such as chemokines (39), the exosome (40, 41), or microRNA (42) facilitate crosstalk between cells in the BM (43, 44) Extracellular matrix (ECM) proteins deposited from cells provides the BM architecture and determine the stiffness of tissue, which affects the cell proliferation and chemosensitivity of leukemia (45). The BM microenvironment has been studied for its role in leukemia support and drug resistance (46, 47). A previous study examined BCR-ABL positive (Ph^+^) (Tom-1, Nalm-27 and Sup-B15) and BCR-ABL negative (Ph^−^) cell lines (REH and Nalm-6) cultured on primary bone marrow stromal cells (BMSC) or osteoblasts (HOB) divided into three populations by relative distance to the supportive layer–S (suspended), phase bright (PB), and phase dim (PD). Out of the three populations, the PD leukemic population planted under the BMSC layer demonstrated increased quiescence; resistance to cytarabine (Ara-C), methotrexate (MTX), and vincristine (VCR); and increased glycolysis (48). This result shows the importance of crosstalk between leukemia and the surrounding microenvironment. One of the most well-known mechanisms of the BM–leukemia cell interaction is the CXCR4/CXCL12 axis (2, 49), yet there are many more surface molecules that are directly associated with adhesion and interaction (50–52). Cell adhesion molecules (CAMs) are cell surface proteins that are specialized for adhesion to other types of cells or the ECM. This review will primarily focus on leukemia-relevant adhesion receptors from four major families of CAMs - cadherin, immunoglobulin superfamily CAM (IgCAM), selectin and integrin (53).

CAMs are single-pass transmembrane proteins with extracellular, transmembrane, and intracellular structures. The extracellular domain of CAMs recognizes specific ligands or counter-receptors, and the intracellular (cytoplasmic) domain translates external stimuli into intracellular signalings, while the transmembrane domain stabilizes the structure of the molecule.

While selectins act as a monomer, cadherins form a homodimer and integrins must a heterodimer in order to be fully functional. Ca^2+^ is required for stabilization of extracellular domain (54–56), as well as in selectins for proper binding to ligands (57). Integrins are dependent and regulated by other divalent cations as well (58, 59). Ca^2+^ binding maintains the folded and inactive conformation of integrins while the heterodimer travels from the Golgi to the cell surface, and integrin undergoes conformational changes upon replacement of Ca^2+^ with Mg^2+^ or Mn^2+^ (58–60).

**Figure 1:**
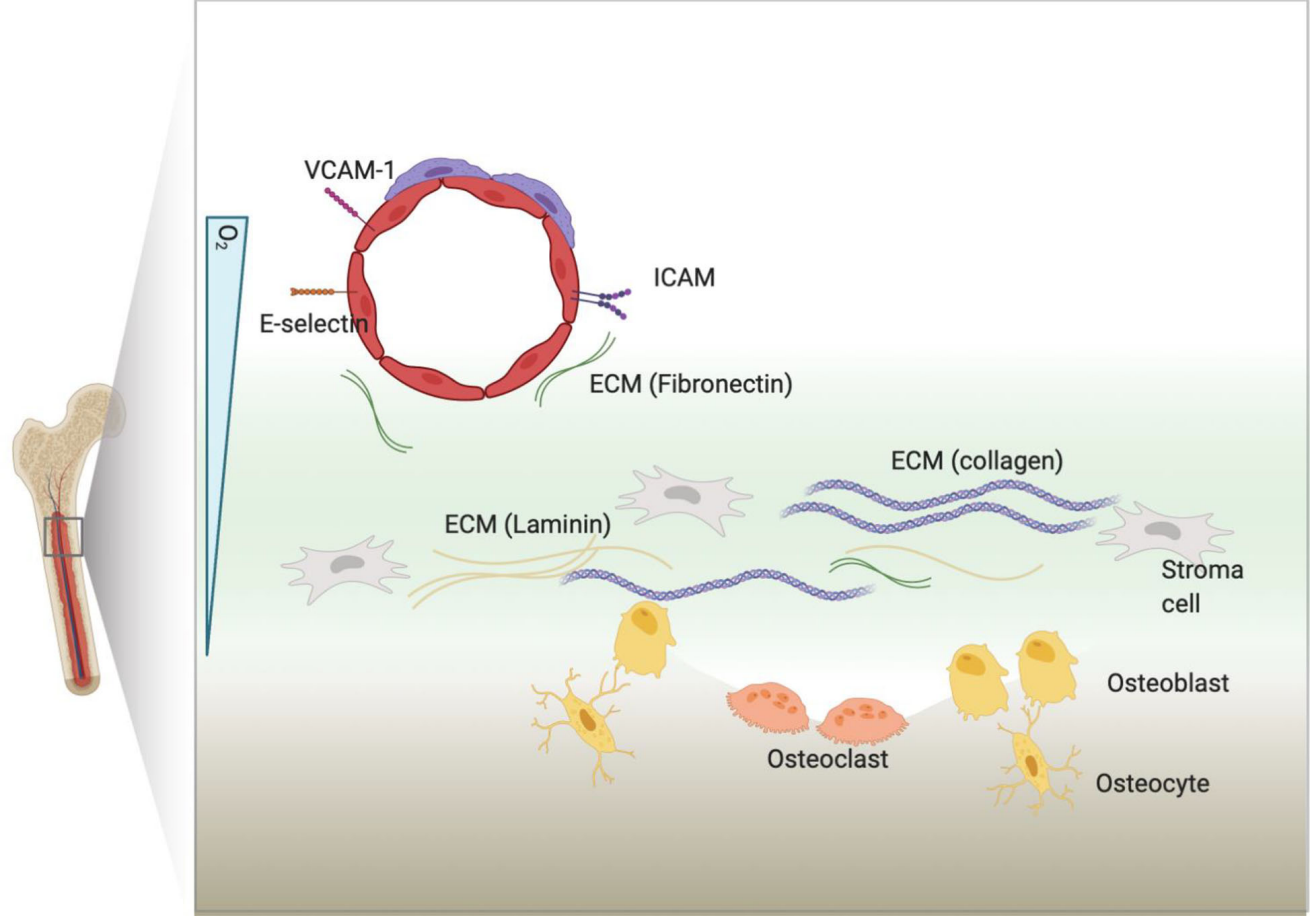
BM microenvironment. BM includes many types of cellular and non-cellular components. Cellular components express ligands or counter receptors, such as VCAM-1 or ICAM, that will bind to CAMs. Cells can also secret extracellular matrix (ECM) proteins that will bind to CAMs.

BM is abundant in binding partners for CAMs as each components of BM discussed above express diverse ligands and secret ECM (61–68) (**Figure 1**). Fibronectin, collagen and laminin secreted in the BM will interact with cellular surface molecules (69, 70). Leukemia-induced BM remodeling can interrupt homeostasis and shift equilibrium towards leukemia progression by overexpressing binding partners for CAMs (71–74). At the same time, leukemia cells can aberrantly express CAM to facilitate surface molecule-mediated interaction with BM microenvironment, thereby inducing cell-adhesion mediated drug resistance (CAM-DR). Recently, the mitochondrial transfer from mesenchymal cells to leukemia has been shown to promote drug resistance in T-ALL, indicating the diverse aspects that BM can modulate to provide leukemia protection (75).

## Cadherins In Cell Adhesion-Mediated Drug Resistance In Leukemia

**Figure 2:**
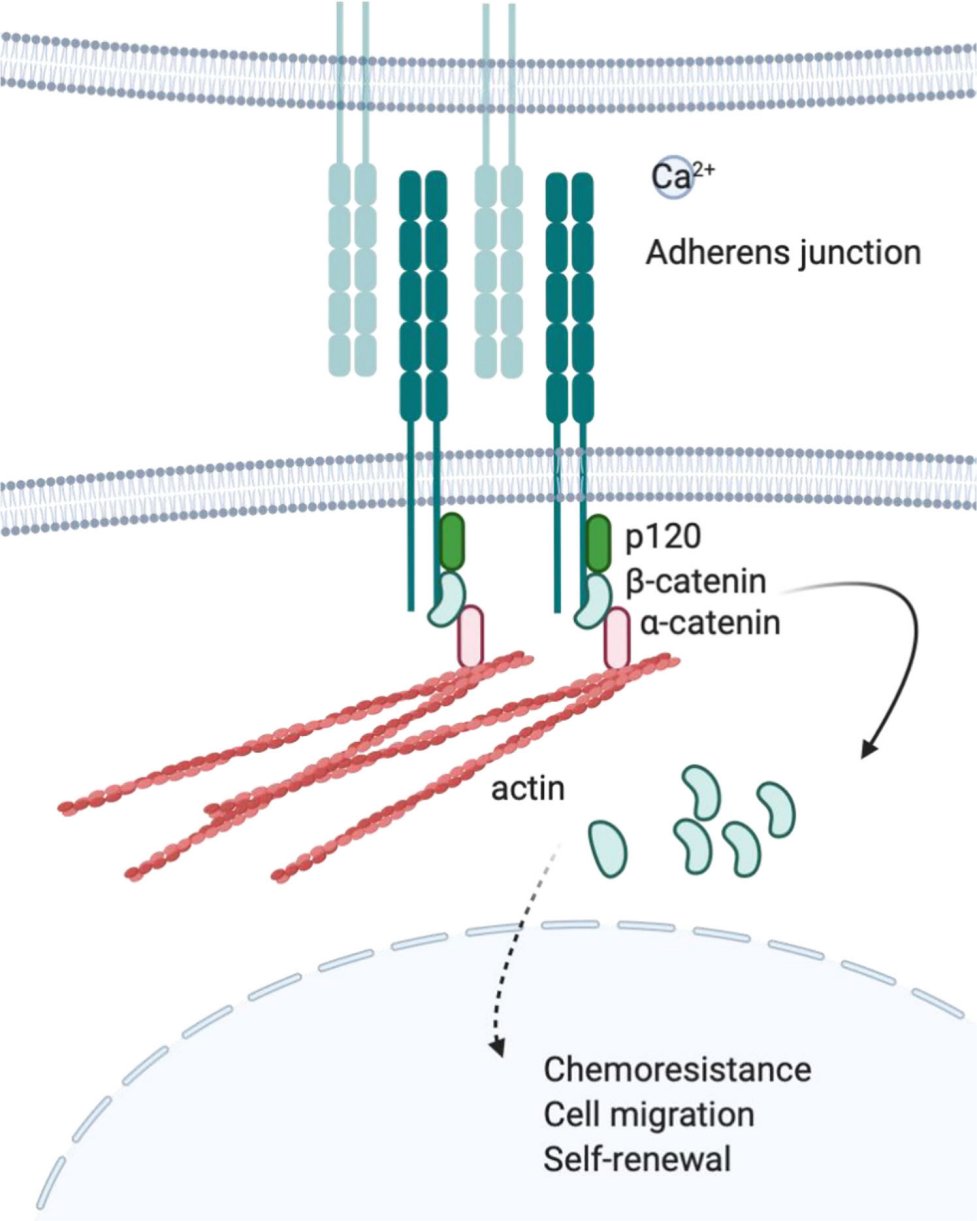
Cadherin and adherens junction. Upon engagement in homotropic manner, cytoplasmic tail of cadherins will bind to actin through p120, β-catenin, and α-catenin protein complex. Cadherin mediated protein complex formation is observed at adherens junction where adjacent cells are connected to each other.

Cadherins are a type of CAM that participates in forming adherent junctions between adjacent cells. Cadherins can be subdivided into several different groups including Type I and Type II classical cadherins (76), desmosomal cadherins (77), proto-cadherins (78), seven-pass transmembrane cadherins, and FAT and Dachsous cadherins (79). The extracellular domain contains cadherin extracellular repeats that exert homotypic Ca^2+^ dependent adhesion, while the intracellular domain binds p120-catenin and β or γ-catenin (80–82). Furthermore, β-catenin will interact with α-catenin, which binds actin filaments (83–85). Adherens junction complexes formed between two cells connect epithelial and endothelial cells (86) (**Figure 2**). E-cadherin, N-cadherin, and P-cadherin are classified as Type I, while VE-cadherins are Type II classical cadherins, which have been well-studied in the context of cancer biology. In metastatic epithelial tumors, the downregulation of E-cadherin compensated by the expression of other cadherins, such as N-cadherin, is a hallmark of the epithelial–mesenchymal transition (EMT). This “cadherin switching” enables tumor cells to acquire a metastatic phenotype that is different from the parental population. Indeed, E-cadherin is considered a tumor suppressor as it inhibits transformation by blocking β-catenin signaling (87). Therefore, dysfunctional or decreased expression of E-cadherin is associated with cancer progression and metastasis. However, cadherin switching is a late event in tumorigenesis and is context-dependent (e.g., exposure to certain soluble factors or interaction with specific ECM proteins) (88, 89).

### VE-CADHERIN

Although leukemia does not necessarily undergo EMT, E-cadherin expression is reduced by hypermethylation of the E-cadherin gene promoter (90, 91), while VE-cadherin and N-cadherin expression contributes to chemoresistance in BCR-ABL^+^ (Ph^+^) ALL and CML (92, 93). VE-cadherin expression along with PECAM-1 expression on ALL enhances the adhesion of leukemia cells to human brain-derived microvasculature endothelial cells (HBMECs) and their migration through the HBMEC monolayer, suggesting a role of VE-cadherin in the central nervous system (CNS) infiltrating leukemia (94). Furthermore, stromal cells upregulate VE-cadherin expression in BCR-ABL+ leukemia cell lines (K562 and SUP-B15) and increase resistance to imatinib by stabilizing β-catenin (95). β-catenin is an important component in cadherin-mediated adhesion as it bridges the cytoplasmic tail of cadherin to the actin cytoskeleton and stabilizes the adherent junction. Since β-catenin binds to transcription factors to initiate transcription, cadherin adhesion is often associated with the activation of Wnt/β-catenin intracellular signaling pathways (96). A subpopulation of the Ph+ B-ALL cell line SUP-B15 presents leukemia stem cells (LSCs) and expresses stem cell markers (CD34, CD38, and c-Kit) and endothelial antigens (Flk-1 and PECAM-1); moreover, LSCs express VE-cadherin after a long-term co-culture on stromal cells (97). The overexpression of VE-cadherin on Ph^+^/VE-cadherin^+^ LSC populations stabilizes β-catenin, maintaining β-catenin as constitutively active and thus promoting self-renewal independent of Wnt signaling. The same group later showed that VE-cadherin regulates apoptosis in Ph^+^ ALL (98).

### N-CADHERIN

Apart from VE-cadherin, N-cadherin is also associated with LSCs. In AML patients treated with a HAD regimen of homoharringtoninetcytosine (HHT), cytarabine (Ara-C), and daunorubicin (DNR), the N-cadherin and Tie2 expressing CD34^+^/CD38^−^/CD123^+^ LSC population presented higher expansion than AML patients who did not receive chemotherapy, indicating that the expression of N-cadherin and Tie2 on AML cells provides a survival benefit against the therapy (99). In addition, the N-cadherin expressing cell line KG-1 and AML patient-derived bone marrow mononuclear cells (BMMNCs) were able to form more colonies compared to an N-cadherin negative control (26). A higher percentage of N-cadherin^+^ cells were shown to remain at the G0/G1 phases compared to N-cadherin^−^ cells and showed higher engraftment in NOD/SCID mice compared to the negative control. Indeed, N-cadherin^+^ cell bearing mice had a significantly shorter survival than the N-cadherin^−^ engrafted mice. This suggests a role of N-cadherin in maintaining the stem cell-like properties and survival of LSCs, which results in relapse. Mesenchymal stromal cell (MSC)-N-cadherin adhesion in CML LSC also provides tyrosine kinase imatinib resistance by stabilizing N-cadherin/β-catenin complex formation and the nuclear translocation of β-catenin in concert with the activation of exogenous Wnt/β-catenin signaling (92). When the N-cadherin-mediated adhesion of CML cells to MSCs was interrupted with anti-N-cadherin short cyclic HAV peptide (NCDH), the CML cells gained sensitivity toward imatinib treatment.

### E-CADHERIN

Another study supported E-cadherin as an important mediator for AML pathogenesis, indicated by stalled differentiation accompanied with high proliferation (100). In this study, carbohydrate-binding protein lectin LecB induced differentiation of the AML cell line THP-1 and increased apoptosis of cells in a dose and time dependent manner. LecB-induced differentiation was mediated by increased autophagy and decreased cellular β-catenin levels, the balance of which is a crucial factor for regulating the differentiation of AML cells. In differentiating cells, LecB was in proximity to the membrane E-cadherin and further promoted the co-localization of E-cadherin and β-catenin. Interestingly, fewer LecB treated THP-1 cells were suspended in the supernatant, suggesting greater adhesion to the cell culture plate, but the direct association of E-cadherin and adhesion was not investigated in this study.

Drugs developed to interrupt the interactions between malignant cells and the microenvironment decreased cadherin expression on malignant cells as a secondary effect without directly targeting cadherin. Recently, an adenosine analogue, Cordycepin, was proposed as an anti-leukemia therapeutic adjuvant. Cordycepin’s anti-leukemic effect in U937 and K562 cells was achieved through the reduced attachment of leukemia cells to MSC by decreasing N-cadherin expression on leukemia cells and vascular cell adhesion molecule-1 (VCAM-1) in MSCs (101). Targeting bone marrow endothelial cells (BMECs) with combretastatin, a microtubule assembly inhibitor, increased AML cell dislodgement from BMECs (102). Combretastatin decreased VCAM-1 and VE-cadherin on BMECs, and dislodged AML cells shifted G0/G1 to G2/M in their cell cycle. A combination treatment of combretastatin and cytotoxic chemotherapy increased induction apoptosis in AML cells. Taken together, cadherins play an important role in leukemia drug resistance. Particularly, chemotherapy-resistant leukemic stem cell (LSC) populations utilize cadherin, which further stabilizes β-catenin and thus activates the expression of genes important for self-renewal.

### TARGETING CADHERINS IN LEUKEMIA

Cadherin inhibitors were developed based on compelling preclinical data and translated into clinical trials. A cyclic pentapeptide ADH-1 against N-cadherin is the most commonly studied cadherin inhibitor in cancer models. Thus far, this pentapeptide has been tested as a single agent or in combination with conventional chemotherapy in patients with N-cadherin expressing solid tumors (103–107). Due to N-cadherin’s role in tumor metastasis, drug resistance and bone marrow homing, N-cadherin has been proposed as a potential target to treat hematological malignancies in patients (108) (**Table 1**).

**Table 1:** Description of cadherin inhibitors.

Drug name	Description	Indication	References
ADH-1	N-cadherin inhibitor	Solid tumors	(104–106)
FX06	Competitive inhibitor of fibrin E1 binding to VE-cadherin	Cardiac reperfusion injury and myocardial infarction	(109, 110)
Celecoxib	COX-2 inhibitor	Evaluated for anti-cancer effects by binding to cadherin-11 and regulating E-cadherin expression	(111–113)

FX06 is a naturally occurring peptide derived from the Bβ_15-42_ sequence of human fibrin that is cleaved and released from the parental fibrin and competitively binds to VE-cadherin (114). FX06 was evaluated in myocardial infarction, yet it failed to show significant baseline benefits compared to a placebo-treated group, although the necrotic core zone significantly decreased (109). FX06 has not been tested in any cancer to date.

Celecoxib is a nonsteroidal anti-inflammatory drug that inhibits prostaglandin-endoperoxide synthase 2, also known as COX-2. Interestingly, the effects of celecoxib were studied to treat calcific aortic valve disease (CAVD) because of their cadherin-11 binding properties (115, 116). Celecoxib was shown to promote anoikis by downregulating E-cadherin in osteosarcoma cell line MG-63 by decreasing PI3K/Akt (117). However, E-cadherin downregulation is a characteristic of EMT in invasive tumors when accompanied with N-cadherin expression; thus celecoxib has been proposed to induce EMT in ovarian cancer (118). Despite different views on celecoxib, there have been more than 100 related clinical trials in the U.S. for cancer patients, including two trials on leukemia and hematological malignancies (119). Celecoxib showed the inhibition of proliferation and survival by downregulating β-catenin in Ph^+^ CML (120), restoring imatinib sensitivity in imatinib-resistant CML (121), and exerting an anti-tumor effect in the HL-60 AML cell line (122), yet its relationship with cadherin-11 is not yet specified. As Wnt signaling has been shown to be an aberrantly upregulated pathway in leukemia (123) and is involved with chemoresistance, this warrants further exploration of celecoxib as a viable therapy to reverse CAM-DR in leukemia.

## Selectins In Cell Adhesion-Mediated Drug Resistance In Leukemia

Selectins (CD62) are single-chain transmembrane glycoproteins that mediate calcium-dependent carbohydrate-binding. There are three major types of selectins: L-selectins are majorly expressed on leukocytes, E-selectins are expressed on endothelial cells, and P-selectins are expressed on activated platelets (124, 125). Selectins share common structures: (1) Calcium-dependent lectin domain, (2) an epidermal growth factor (EGF)-like domain, (3) a variably-sized repeated region, (4) a transmembrane domain, and (5) a cytoplasmic domain (126). The main function of selectins is to facilitate leukocyte tethering and rolling along endothelial cells, which is an initial step of the transmigration of leukocytes through the endothelial barrier. Briefly, free floating cells expressing selectin ligands, such as P-selectin glycoprotein ligand-1 (PSGL-1), bind to P-selectin expressing endothelial cells. Upon engagement, the leukocyte movement will slow down and the cells remain in proximity to the vessel wall, while integrin-ICAM/VCAM-1 interactions and other cytokine-mediated tight adhesions between leukocytes and endothelial cells strengthen the binding for transmigration. As a result, leukocytes can travel to distant sites of inflammation, and hematopoietic stem cells (HSCs) can home into the bone marrow (127).

Because the endothelium binding of leukocytes is a prerequisite of metastasis, selectins are well-known to be involved in cancer progression (128, 129). E-selectin expressed on the endothelium is the primary source of binding partners for leukocytes, and T-ALL cells were not able to adhere to interleukin-2 activated human umbilical vein endothelial cells (HUVEC) upon E-selectin inhibition with a monoclonal antibody (130). E-selectin expression in the BM vascular niche has been proposed to be regulated by Runt-related (RUNX) transcription factor, and RUNX silencing was shown to downregulate E-selectin expression and lead a subsequent decrease in AML engraftment in the BM in mice (131).

Leukemia cells interact with E-selectin through various ligands such as CD43, CD44, and PSGL-1. Particularly, myeloblasts favor PSGL-1 for interactions with endothelial selectins, while lymphoblasts express less PSGL-1. PSGL-1 expressed on the surface of the leukemia cells can bind to P-selectin along with E-selectin on endothelial cells (132). In contrast, lymphoblasts mainly use CD43 and/or CD44 to bind to endothelial selectins (133). Therefore, even though specific ligands are preferentially used in different cells, this does not mean that other molecules are less crucial in conferring CAM-DR. Indeed, myeloblasts use PSGL-1, CD44, and CD43 to various extents during E-selectin binding. Therefore, different patients show different profiles of E-selectin ligand expression levels. Interestingly, nilotinib treatment upregulated E-selectin, ICAM-1, and VCAM-1 expression on human endothelial cells (134), which may result in the increased adherence of leukemia cells to E-selectin and the evasion of chemotherapy-induced cytotoxicity. In fact, a high baseline level of soluble E-selectin along with VEGF, PAI-1, and low initial soluble ICAM-1 were proposed as prognostic factors for poor outcomes in pediatric ALL (135). Leukemia itself can express selectins on the surface to promote migration and progression. Human BCR-ABL1 (p210) retrovirus transduced murine leukemia expressed integrin subunit alpha-6 and L-selectin, which were used to metastasize into the central nervous system, predominantly in meninges (136).

### Targeting Selectin in Cancer

Currently, selectin inhibition is actively being translated into leukemia treatments (**Table 2**). GMI-1271 (Uproleselan), an E-selectin antagonist, is intended to inhibit E-selectin expression on endothelial cells so that E-selectin-mediated drug resistance in leukemia can be prevented. Preclinical investigations of GMI-1271 in multiple myeloma (MM) showed the sensitization of E-selectin ligand rich in MM toward bortezomib (142). GMI-1359, a dual E-selectin and CXCR4 inhibitor, significantly decreased bone metastasis, synergized the docetaxel effect in prostate cancer cells (137), and sensitized MM toward carfilzomib (138). Currently, GMI-1271 is being investigated for its safety and efficacy in AML patients (**Table 3**). Crizanlizumab (Adakveo) is a monoclonal antibody against P-selectin, which is expressed on the surface of the activated endothelium and platelets. Crizanlizumab is used to reduce vaso-occlusive crises (VOC) in adult and pediatric patients with sickle cell disease (SCD). Clinical trials are open for dose confirmation and safety in both adult and pediatric SCD patients to evaluate the safety and efficacy on SCD-related complications along with a combination study of myelofibrosis with ruxolitinib. YSPSL (rPSGL-lg), a P-selectin glycoprotein ligand IgG fusion protein, binds to P-selectin and was evaluated in ischemia-reperfusion injury, liver disease, and kidney functions but has not been tested in cancer (143).

**Table 2:** Description of selectin inhibitors.

Drug name	Description	indication	References
GMI-1271 (Uproleselan)	E-selectin inhibitor	Small molecule inhibitor against E-selectin on endothelial cells to treat AML and potentially other hematologic cancers	(28)
GMI-1359	E-selectin/CXCR4 dual inhibitor	Targeting E-selectin and CXCR4 to reduce tumor metastasis to bone marrow	(137, 138)
Crizanlizumab (Adakveo)	Monoclonal antibody against P-selectin	Reduction of vaso-occlusive crises in sickle cell disease patients	(139)
YSPSL (rPSGL-lg)	Recombinant P-selectin glycoprotein ligand IgG fusion protein	Myocardial infarction, red blood cell disorders, anemia, transplant, ischemic-reperfusion injury	(140, 141)

**Table 3:** Clinical trials for selectin inhibitors in leukemia.

Drug	Target	Condition or disease	Phase	Intervention/treatment	References
GMI-1271	E-selectin	AML	I/II	Evaluation of GMI-1271 treatment combined with mitoxantrone, etoposide, cytarabine and idarubicin in AML patients	NCT02306291
GMI-1271	E-selectin	Relapsed/ refractory AML	III	Efficacy of uproleselan (GMI–1271) in combination with mitoxantrone etoposide and cytarabine (MEC), or fludarabine, cytarabine and idarubicin (FAI) in relapsed/refractory AML patients	NCT03616470
GMI-1271	E-selectin	AML (adults 60 years and older)	II/III	Evaluation of uproleselan combined with cytarabine or daunorubicin in older AML patients receiving intensive induction chemotherapy.	NCT03701308

GMI-1271 is currently the only selectin inhibitor in clinical trials and is being investigated for its safety and efficacy in AML patients (**Table 3**). There is increasing evidence in support of the importance of targeting the BM microenvironment due to its role in therapy resistance (144–146). In these clinical trials, GMI-1271 will be administered in combination with existing chemotherapies, which highlights how targeting both the microenvironment and leukemia cells may be necessary in order to ameliorate CAM-DR.

## Integrins in Cell Adhesion-Mediated Drug Resistance

**Figure 3:**
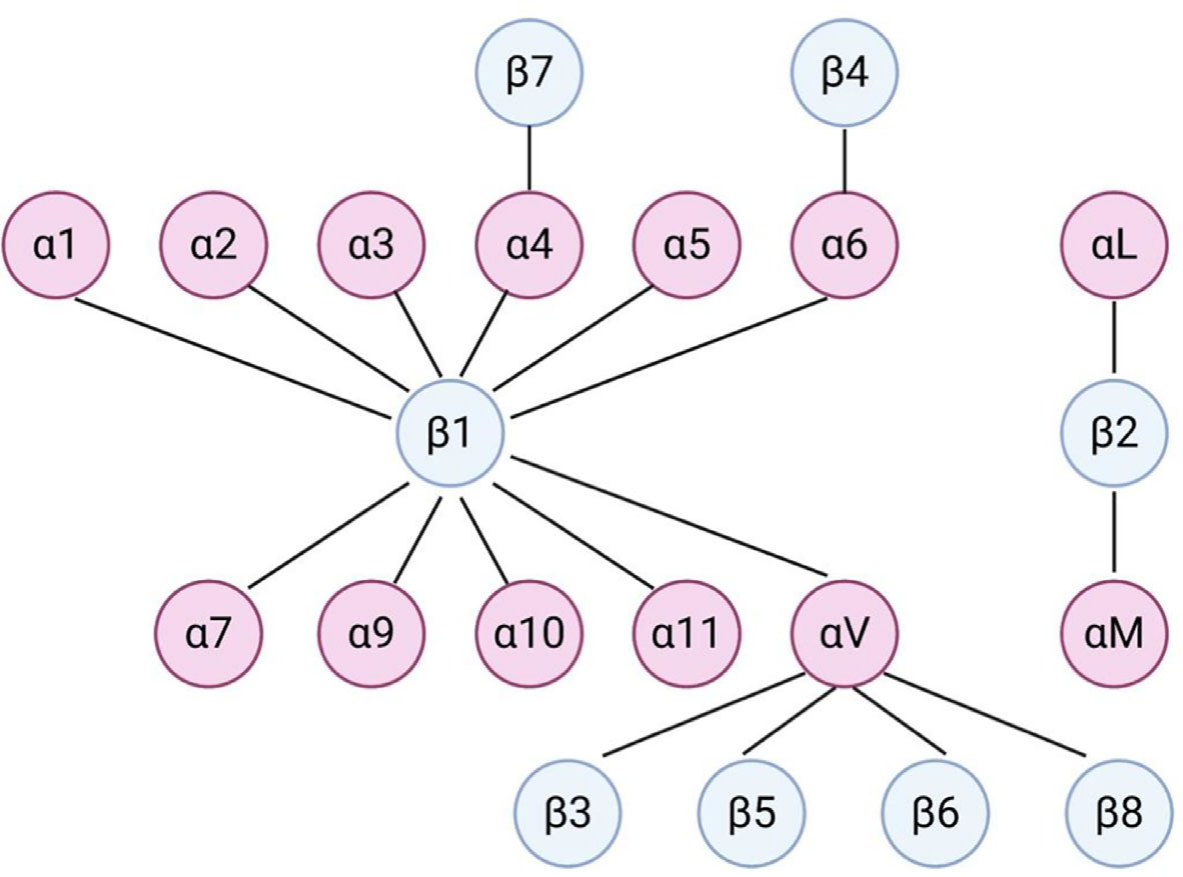
Dimerization of integrins in leukemia. Dimerization of α and β integrins forms a functional heterodimer unit. This figure does not represent a comprehensive list of integrin heterodimers but rather includes integrins described by currently available publications in the field of leukemia, which are reviewed in this review.

**Figure 4:**
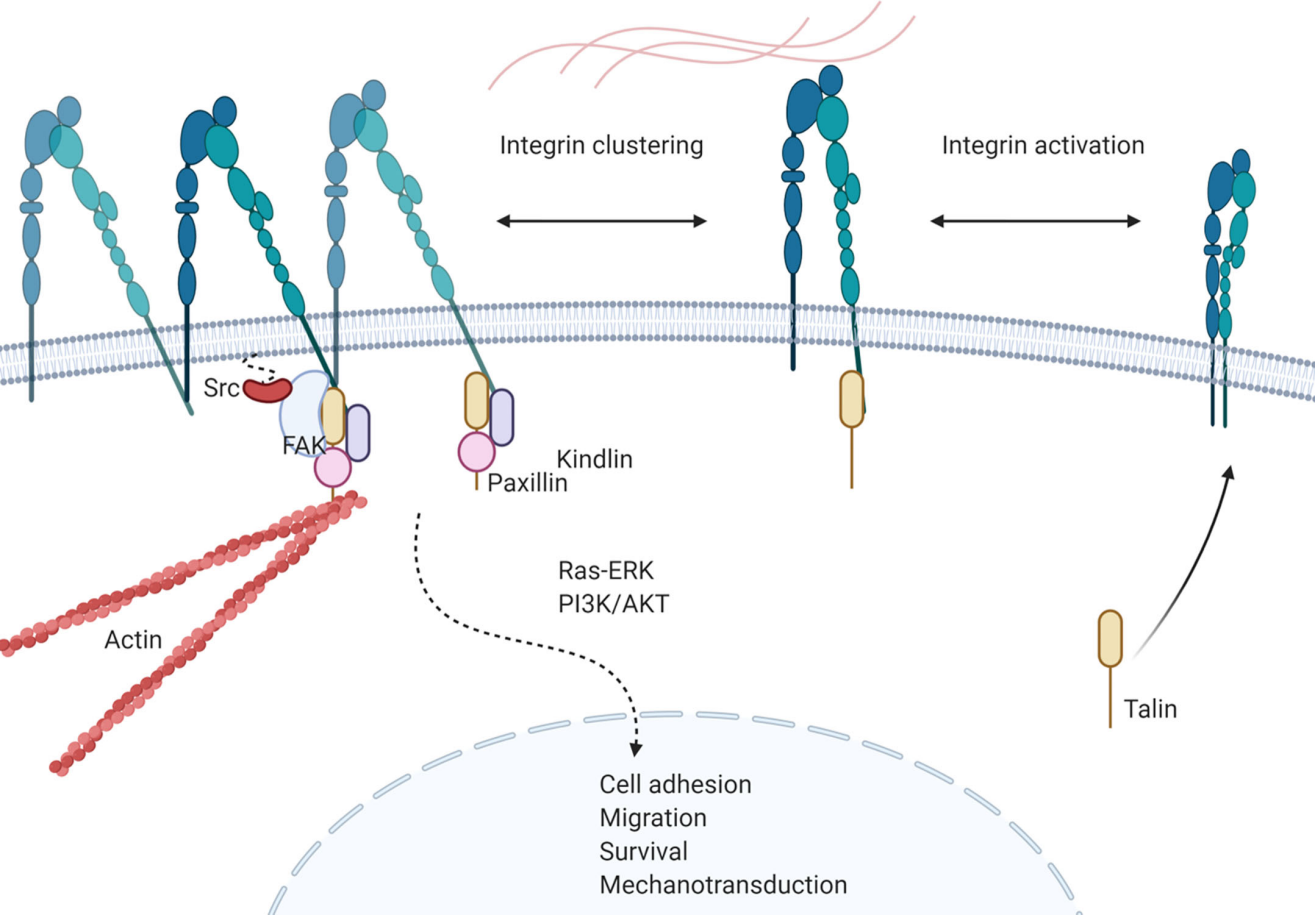
Integrin signaling in leukemia. Talin binding to cytoplasmic tail of β-subunit activates integrin heterodimer and increases affinity of the complex towards ligands. Activation of integrin is followed by conformational change of the heterodimer and separation of cytoplasmic tails of each subunit, allowing recruitment of proteins. Recruited proteins, such as kindlin, paxillin, FAK and Src forms a protein complex that initiates integrin mediated intracellular signaling that results in cell adhesion, migration, survival and mechanotransduction of leukemia cells.

Integrins are calcium independent type-I transmembrane proteins with a shared structure of the extracellular domain, the transmembrane domain, and the cytoplasmic domain. Composed of 18 alpha (α) and 8 beta (β) subunits, integrins participate in cell–cell or cell–ECM adhesion. To date, 24 integrin heterodimers with different combinations of α and β subunits are known and can interact with their ligands in arginine–glycine–aspartic acid (RGD) sequence dependent and independent manners (147, 148) (**Figure 3**). **Figure 3** does not represent a comprehensive list of integrin heterodimers but rather includes integrins described by currently available publications in the field of leukemia, which are reviewed in this review. During “inside-out” signaling, binding of talin to the cytoplasmic tail of the β subunit increases integrins’ affinity toward their ligands by undergoing conformational changes from low-affinity to high-affinity state (149, 150). On the other hand, binding of a ligand or a counter-receptor to a specific domain on theα subunit or the αβ heterodimer results in spatial separation of cytoplasmic tails of α and β subunits. This event allows adaptor proteins such as talin and vinculin to engage with β tail and associate with the cytoskeleton and form a protein complex called focal adhesion and integrin clustering (151–153) (**Figure 4**). Integrins can be internalized and recycled, thus controlling availability of integrin heterodimers on the plasma membrane (148, 154). These processes will translate external stimuli and environmental cues into intracellular signaling and mediate adhesion, cell spreading, migration, proliferation and survival in cells. Integrin-dependent adhesion to ECM can convert mechanical forces into biochemical signals, allowing cells to recognize biophysical properties of given BM microenvironment (155). Few studies analyze redundancy between integrins in CAM-DR, and how it might affect integrin targeting. Future studies will need to address this gap of knowledge. Here, we summarized integrins by name.

### INTEGRIN α 1 (CD49a)

Integrin alpha 1 subunit forms a heterodimer with the integrin beta 1 subunit to form α_1_β_1_, which binds to collagen and laminin (156, 157). α_1_β_1_ is also called very late antigen 1 (VLA-1) because it is expressed on the surface of long-term activated T cells (158). VLA-1 mediates the adhesion of intraepithelial lymphocytes (IELs), such as the CD8^+^ T cells found in the intestinal epithelium, to collagen (159). α_1_ was also suggested to be a potential marker for stromal cells and is expressed in more than 80% of human derived non-transformed bone marrow stroma cells. In this study, only α_1_ expressing stroma precursors was able to give rise to colony-forming unit-fibroblasts (CFU-F) compared to the α_1_ negative subgroup of the stromal population, suggesting α_1_ as a marker for stromal precursor cells (160). Embryonic fibroblasts derived from α_1_ deleted mice were not able to spread or migrate to either collagen IV or laminin, suggesting their importance in cell spreading and migration (161). However, the role of VLA-1 in CAM-DR in leukemia is still unknown.

### INTEGRIN α 2 (CD49b)

Integrin alpha 2 forms a heterodimer with the beta 1 subunit to form a VLA-2 molecule that binds to collagen and laminin (162). A real-time quantitative polymerase chain reaction study on 134 *de novo* AML patients revealed higher ITGA2 expression in AML patients compared to the 33 normal controls (163). Moreover, ITGA2^high^ patients had significantly lower complete remission (CR) rates and a shorter overall survival compared to the ITGA2^low^ groups. ITGA2 expression decreased significantly in the patients who achieved CR but increased again in relapsed patients, suggesting that ITGA2 is a marker for a poor prognosis in AML. The α_2_β_1_ mediated adhesion of the T-ALL cell lines Jurkat and HSB-2 and the primary T-ALL blasts toward collagen I decreased doxorubicin induced apoptosis (164). α_2_β_1_ mediated adhesion activates the MAPK/ERK pathway, which inhibits the doxorubicin-induced activation of c-Jun N-terminal kinase (JNK) and maintains the pro-survival protein Bcl-2 family member Mcl-1. The same group extended α_2_β_1-_collagen mediated doxorubicin resistance in the AML cell lines HL-60 and U937 (165). In AML, collagen binding through α_2_β_1_ inhibited the activation of the pro-apoptotic protein Rac1, thereby preventing Rac1 induced DNA damage.

### INTEGRIN α 3 (CD49c)

VLA-3 interacts with ligands in both an RGD-dependent and RGD-independent manner. VLA-3 mediated adhesion to fibronectin requires RGD-motif recognition, whereas binding to collagen and laminin does not require an RGD-motif in the ligands (166). VLA-3 binding to fibronectin increased in the presence of the Mg^2+^ and Mn^2+^−divalent cations required for integrin activation, whereas binding to collagen and laminin was less affected. Integrin-α_3_ was identified as a marker for long-term repopulating hematopoietic stem cells (LT-HSCs) that expand from CD34+ human cord blood cells and retain their self-renewal ability with a long-term engraftment pattern compared to short term HSCs (SC-HSCs) (167). Furthermore, ITGA3 knockdown with short hairpin RNA against *ITGA3* did not affect the stemness of the cells but decreased the long-term reconstitution ability in NSG mice. However, the specific role of VLA-3 in CAM-DR in leukemia is still unclear.

### INTEGRIN α 4 (CD49d)

Integrin α_4_ binds with either the β_1_ or β_7_ subunit to form α_4_β_1_ or α_4_β_7_ heterodimer. Integrin α_4_ exerts physiological effects including cell adhesion and migration, while triggering intracellular signaling, thereby indicating the promotion of leukemia cell drug resistance and survival. Integrin-α_4_ knockout mice are embryonically lethal (168). There are specific relationships between integrin α_4_, epigenetics, metabolism, and cell surface markers. Histone deacetylase inhibitor treatment may downregulate VLA-4 for various AML cell lines, primary patient samples, and normal hematopoietic stem cells (169). The expression of G9a, a histone methyltransferase related to gene silencing, correlates with integrin α_4_ expression in pediatric B- and T-ALL. Furthermore, G9a depletion or inhibition with BIX01294 was shown to abrogate the ability of ALL cell migration towards the endothelial monolayer (170). Moreover, it was recently reported that tetraspanin (CD9)^+^ B-ALL is associated with a poor prognosis. In this study, CD9 physically interacted with VLA-4 and mediated the affinity to VCAM-1. CD9 inhibition interrupted the leukemia–stroma interactions and sensitized B-ALL cells to chemotherapy (171). CD98 has been shown to interact with the cytoplasmic domains of β1 and β3 and mediate the adhesive signaling of integrin α4/VCAM-1 in AML (172). The redox modulation of adjacent thiols in VLA-4 by AS101, an IL-1β converting enzyme, restored the chemosensitivity of AML cells by decreasing PI3K/Akt/Bcl2 signaling (173). It has been demonstrated that integrin α4 and α5 are involved in Jurkat T-ALL adhesion-independent chemoresistance (174). Our studies also showed that both deletion and inhibition with natalizumab of integrin α4 sensitize primary B-ALL cells to chemotherapy (175). Furthermore, the CD49d antisense drug ATL1102 efficiently downregulated the CD49d mRNA level of B-ALL *in vitro* (176). Anti–VLA-4 antibodies (SG/73, SG/17) were shown to increase chemosensitivity in human AML cells and eradicate minimal residual disease (MRD) in experimental mice when combined with chemotherapy (6). Integrin α4 has been shown to be a prognostic marker of poor overall survival in B-ALL (175). Interestingly, a report from the Children’s Oncology Group found that high VLA-4 expression is associated with a better clinical outcome in pediatric AML and is an independent predictor of relapse (177). Similar results were found in a study of the Southwest Oncology Group trials (178).

### INTEGRIN α 5 (CD49e)

The integrin α_5_ subunit can dimerize with integrin β_1_ to form α_5_β_1_ (VLA-5), which binds to the RGD sequence in fibronectin (179). Both murine and CD34^+^ human HSCs were shown to bind to a recombinant peptide of the VLA-5 binding RGD-motif of fibronectin *in vitro* (180). The preincubation of B6.*Hbb^d^/Hbb^d^, Gpi-1^a^/Gpi-1^a^* mice-derived BM cells incubated with the fibronectin binding domain including the peptide CH-296 decreased the engraftment of BM cells in recipient mice, and the intravenous injection of the CH-296 peptide caused an increase in the percentage of progenitor cells in the spleen, suggesting the importance of VLA-5 in HSC engraftment in the BM. VLA-5 has been suggested as a therapeutic target in leukemia. A subset of ALL includes an alteration in the *IKAROS* gene, which is correlated with a poor prognosis. The exon 5 deletion of *Ikzf1* in pre-B cells arrests the cells in an “adherent phase”, where survival and proliferation depend on stable adhesion to the stroma with increased Erk1-2 MAPK activity (181). The expression of the dominant-negative Ikaros isoform IK6 in the T-ALL (Jurkat) and B-ALL cell lines (RS4;11, Nalm6) lifted the transcription suppression of FUT4, which fucosylates α_5_β_1_ on leukemia cells and tightens the adhesion of ALL cells to fibronectin in the ECM. This increased adhesion was achieved *via* activation of the FAK/Akt pathway upon Lewis X (Le^X^, CD15, or SSEA-1) modification of α_5_β_1_ (182). In U937 and blasts from AML patients, α_4_β_1_ and α_5_β_1_ mediated the adhesion of cells to fibronectin, and the addition of the Wnt antagonist sFRP induced resistance to daunorubicin 16407823 (183). Both adhesion and the Wnt pathway contribute to chemoresistance in AML and require the activation of glycogen synthase kinase 3β (GSK3β). Upon serum starvation of AML U937, VLA-5 binding to fibronectin regulates specific pro-survival functions through the activation of GSK3β (184). VLA-5 inhibition with an anti-integrin α_5_ antibody sufficiently decreased adhesion of the Ph^+^ ALL cell line SUP-B15 to fibronectin, while a combination of VLA-5 inhibition with imatinib synergistically increased apoptosis in SUP-B15 cells *in vitro* (185). Furthermore, the inhibition of VLA-5 with disintegrin, an antibody, or knocking down integrin-α_5_ impaired the engraftment of SUP-B15 cells in immunodeficient mice. A combination of integrin-α_5_ inhibition with the FAK inhibitor TAE226 prolonged the survival of SUP-B15 engrafted mice, suggesting that the inhibition of VLA-5 combined with conventional chemotherapy may improve the outcome for Ph^+^ ALL patients.

### INTEGRIN α 6 (CD49f, VLA6)

Integrin α_6_ dimerizes with β_1_ to form VLA-6 (186) or with β_4_ to form α_6_β_4,_ which is also known as TSP-180 (187). α_6_ has been suggested to be a biomarker for minimal residual disease since it is expressed on pre-B-ALL at diagnosis, and the signal is preserved or expressed with a higher intensity after therapy (10, 188). α_6_ was found to be expressed significantly more strongly not only in relapsed B-ALL but also in ecotropic viral integration site-1 positive (EVI1^+^) AML cases. In this study, the drug sensitivity of EVI1 AML cells was restored after the inhibition of integrin α_6_ (189). Functionally, α_6_ is suggested to play an important role in the chemoresistance and metastasis of leukemia cells. EVI1^+^ AML cell lines and primary cells were able to bind to laminin better than cell lines with low EVI1. This adhesion is specifically mediated by ITGA6 and ITGB4 expression on EVI1 AML cells, and small-hairpin RNA against EVI1 decreased the expression of ITGA6 and ITGB4. Moreover, the inhibition of ITGA6 or ITGB4 with neutralizing antibodies restored chemosensitivity against Ara-C in EVI^+^ AML cells. In another study, α_6_ on the surface of ALL was shown to facilitate the invasion of ALL cells into the central nervous system by binding to laminin during the process of migration toward the cerebrospinal fluid (190).

Since a high expression of integrin α6 was found on day 29 of an MRD test on B-ALL in the Children’s Oncology Group (COG) P9906 clinical trial, we proposed the drug resistance role of integrin α6. Firstly, we showed that the integrin α6 blockade de-adhered the B-ALL cell from laminin-1 and OP9 stromal cells. Secondly, P5G10, an anti-integrin α6 antibody, in combination with chemotherapy, prolonged the survival of B-ALL xenograft mice. Thirdly, integrin α6 deletion induced apoptosis of B-ALL cells involving Src signaling (191). Recently, it has been shown that the inhibition of integrin-α_6_ is correlated with decreased cell surface deformability using single-beam acoustic tweezers, while no changes in inhibition were shown for integrin α4 (192).

### INTEGRIN α 7 (ITGA7)

Integrin-α_7_ binds with β_1_, which is expressed on skeletal and cardiac muscle (193–196). *ITGA7* was more significantly increased in AML patients with granulocytic sarcoma (GS) compared to patients with GS. Furthermore, integrin-α_7_ mediated the phosphorylation of ERK in the surface integrin-α_7_ expressing AML cell lines PL21 and THP1, thus promoting the proliferation of these cells (197). ITGA7 also has been suggested to be a biomarker for AML patients as ITGA7 expression in AML patients was significantly increased compared to the control and correlated with a poorer prognosis. Patients with either high ITGA7 mRNA or protein expression had a shorter event-free survival (EFS) and overall survival (OS) compared to low ITAG7 patients (198).

### INTEGRIN α 9 (ITGA9)

Integrin-α_9_ dimerizes with the β_1_ subunit to form an α_9_β_1_ that is distributed in the airway epithelium, squamous epithelium, smooth and skeletal muscle, and hepatocytes (199). α_9_β_1_ recognizes tenascin-C (200), osteopontin (201), and VCAM-1 (202). While α_9_β_1_ shares nearly 40% of its amino-acid sequence homology with integrin-α_4,_ both have distinct functions. Integrin-α_9_ knockout mice develop bilateral chylothorax and die 6 to 12 days after birth due to respiratory failure (203). The roles of integrin-α_9_ in the context of leukemia have not been elucidated. Recently, the dual inhibition of α_4_β_1_ and α_9_β_1_ with BOP {[N-(benzenesulfonyl)-L-prolyl-L-O-(1-pyrrolidinylcarbonyl]tyrosine, a small molecule antagonist against integrin α_4_β_1_ and α_9_β_1_} demonstrated the successful HSC mobilization potential from the bone marrow to the peripheral blood. While a single dose of BOP increased the mobilization of huCD45+CD34+ cells by about 2 fold compared to the saline control, a combination of BOP with the CXCR4 inhibitor AMD3100 increased the mobilization of HSC by three fold (204). This result suggests that α_9_β_1_, concomitantly with α_4_β_1_, is involved in the integrin mediated adhesion of HSCs in the bone marrow. Indeed, CD34^+^/CD133^+^ hematopoietic stem and progenitor cells (HSPCs) expressed α_9_ transcripts and α_9_β_1_ on the surface. Integrin-α_9_ mediates the adhesion of CD34^+^ cells to osteoblasts, and the addition of functional blocking antibody against α_9_β_1_ and Y9A2 significantly decreased the proliferation and differentiation of CD34+ HSPC cells (205).

### INTEGRIN α L (CD11a, LFA-1)

Integrin-α_L_ dimerized with a β_2_ subunit is called lymphocyte function-associated antigen-1 (LFA-1). In an early study using T cells derived from leukocyte adhesion deficient (LAD) patients with genetic defects in β_2_ showed a decreased expression of LFA-1 and LFA-1 LAD derived T cells still bound to endothelial cells similar to normal T cells *via* complementary binding through VLA-4, but their transmigration through the endothelial layer of LAD derived T cells was significantly decreased (206). In hematological malignancies, T-cell neoplasms, including T-ALL, almost always express LFA-1, while LFA-1 expression in lymphoma and B-cell neoplasms, including T-ALL, CLL, HCL, and SLL, vary between patients (207, 208). LFA-1 on the T-ALL cell line (Sup T1 and Jurkat) and primary T-ALL were shown to play a critical role in binding T-ALL cells to the BM stroma (HS-5 and patient derived BM) and regulating the survival of T-ALL cells (209).

### INTEGRIN α M (CD11b)

The integrin-α_M_β_2_ heterodimer is called Mac-1 and is known to bind to fibrinogen (210), platelet factor 4 (211), and ICAM-1 (212). The expression of Mac-1 was suggested to be a biomarker for a poor prognosis (213). The Mac-1 mediated adhesion of U937 and HL-60 cells to plastic was suggested to elicit a survival benefit in leukemia cells treated with phorbol ester, and these Mac-1 mediated adherent cells are susceptible to undergo anoikis when forced to be de-adhered, suggesting adhesion dependent survival.

### INTEGRIN α V (CD51, VNRA, MSK8)

Integrin-α_V_ can dimerize with β_1_, β_3_, β_5_, β_6_, and β_8_. Heterodimers including α_V_ can bind to fibronectin (214) and vitronectin (215, 216). In AML, α_V_β_3_ is suggested to cooperate with the fibroblast growth factor receptor (fgf-R) to increase proliferation, especially the subset of AML that has Hox-overexpression induced by MLL fusion protein (217). In this study, the *MLL-ELL* transduction of primary murine bone marrow cells increased the expression of β_3_ integrin *via* HoxA10, and the α_V_β_3_-mediated adhesion of cells to vitronectin increased Syk, Pak1, and Fak1. α_V_β_3_ activity was reversed through the β_-_catenin and Cdx4 dependent decrease in *ITGB3* promoter activity upon fgf-R inhibition in these cells.

### INTEGRIN β1 (CD29)

Integrin β1 is the most common beta subunit heterodimer partner for integrin alpha subunits (218). In cancer, upregulated expression of β1 is indicative of a poor prognosis (219) and plays roles in chemoresistance by binding to ligands and eliciting downstream signaling events. Berrazouane et al. reported that β1 promotes chemoresistance in T-ALL primary blasts *via* ABC transporter-mediated doxorubicin efflux and the downstream activation of PYK2 (220). Integrin α2β1 binds to collagen and upregulates ABCC1 *via* the ERK/MAPK pathways to modulate efflux (221). Similarly, collagen-binding β1 integrins contribute to doxorubicin resistance in AML by reducing DNA damage through Rac1 inhibition (222).

β1 also has roles in apoptosis inhibition. Estrugo et al. demonstrated that leukemia cell lines HL60 and Jurkat adhere to β_1_ integrin ligands fibronectin, laminin, or collagen-1 and are protected from radiation, Ara-C, and FasL-induced apoptosis (223). These β_1_ integrin-mediated cell-matrix interactions inhibit procaspase-8 activation *via* the PI3K/AKT pathway. Additionally, β_1_ integrin ligation to fibronectin impairs both procaspase-3 and procaspase-9 activation associated with the intrinsic apoptotic pathway.

The tetraspanin superfamily is known to be associated with the activation, ligand binding, and inside-out signaling of β1 integrins and can promote cancer cell survival (224, 225). When β_1_ is expressed with tetraspanin CD82, chemoresistance is promoted by increasing PKCα activation and the downstream clustering of β_1_ integrin, leading to AML cell survival *via* the activation of p38 MAPK for DNA damage repair (226). In summary, integrin β_1_ is implicated in the chemoresistance of leukemia *via* chemotherapy efflux, intracellular signaling, and apoptosis inhibition.

### INTEGRIN β3

It has been suggested that integrin β_3_ may have functional redundancies with β_1_ integrins (227). In AML, β_3_-mediated signaling is required for leukemogenesis and leukemia survival (228), in part through SYK activation. To date, the function of β_3_ in the chemoresistance in leukemia has not been studied. In hepatocellular carcinoma, the upregulation of Galectin-1, which elevates α_V_β_3_expression, was found to activate the PI3K/AKT pathway and is correlated with a poor sorafenib response (229). The antagonism of β_3_ with cilengitide in M2 macrophages led to the promotion of tumor cells, and the loss of integrin β_3_ signaling promoted an immunosuppressive tumor environment (230). Clearly, β_3_ signaling is important in drug response and cancer progression, which may be grounds for similar studies on leukemia.

### INTEGRIN β7

Integrin β7 is present on lymphocytes as a subunit of the α4β7 heterodimer and mediates binding to fibronectin, VCAM-1 (231), and mucosal addressin cell adhesion molecule 1 (MAdCAM-1) (232). α4β7 is less well studied in the context of leukemogenesis and drug resistance and is mainly involved with lymphocyte homing and trafficking. In hematopoietic progenitor cells, α4β7 and MAdCAM-1 contribute to the recruitment of cells into the bone marrow following transplantation, and the inhibition of MAdCAM-1 significantly reduces homing (233). For blood cancers, it has been suggested that the expression of α4β7 plays a role in the leukemic evolution of T cell lymphoblastic lymphomas and the dissemination of lymphoma cells to VCAM-1-positive vascular spaces (234). In T cell leukemias with gastrointestinal involvement, it was found that the expression of α4β7 is linked with homing to MAdCAM-1 on endothelial cells in the intestinal mucosa (235). In summary, while α4β7 may not be involved with leukemogenesis, its roles in lymphocyte homing have effects on the progression of leukemia in different organs.

## Targeting Integrins

The preclinical evaluation of integrin inhibition has suggested promising results for the sensitization of leukemia cells to chemotherapy. Knocking out *ITGA4* restored the sensitivity of BCR-ABL^+^ murine leukemia toward nilotinib (NTB), and a blockade of integrin alpha 4 with a monoclonal antibody sensitized primary B-ALL engrafted xenograft mice to chemotherapy (175). Since the β1 (CD29) subunit dimerizes with many different α units, blockade of β1 is an attractive target for leukemia therapy. In T-ALL, the β1 blockade with β1 specific antibody AIIB2 inhibited cell-matrix interactions and decreased the Matrigel effect on T-ALL colony formation. Furthermore, AIIB2 in combination with doxorubicin significantly prolonged the survival of CEM xenograft mouse models (220). OS2966, a humanized monoclonal antibody, will be used in a phase I clinical trial for glioblastoma and may also have efficacy in the treatment of hematological malignancies by targeting multiple integrins on leukemia cells and the surrounding microenvironment (236). As for other integrins, targeting the active form of the integrin β7 subunit, specifically the MMG49 epitope in the N-terminal region of active β7, showed multiple anti-myeloma effects *in vivo* without damaging normal hematopoietic cells (237). The efficacy of chimeric antigen receptor (CAR) T cells against αvβ3 in melanoma and αvβ6 in ovarian, breast, and pancreatic xenograft mice models has also been evaluated (238, 239) for integrin targeting CAR T therapy in hematological malignancies.

Despite the compelling *in vitro* and *in vivo* anti-tumor effects of integrin blockades in tumor models, the preclinical evaluation of integrin targeting has not yet been successfully translated into a clinical platform. Several clinical trials evaluating integrin inhibition in solid tumors were terminated due to infusion-related reactions and non-significant anti-tumor activity (NCT00915278) (240), insufficient clinical data (NCT00684996), or low enrollment (NCT00675428). A phase II trial of abituzumab (EMD 525797) targeting αv in combination with cetuximab and FOLFIRI in metastatic colorectal cancer is expected to be completed by August 2021 (NCT03688230).

Integrin targeting is useful for the detection of cancers, and many clinical trials target integrin in the CT/PET imaging of cancer patients. A novel radiotracer 99mTc-RWY detecting integrin alpha 6 is in an early phase I clinical trial for SPECT imaging in breast cancer (NCT04289532), and the safety of the radiotracer and potential clinical applications are being evaluated. Likewise, many other types of integrin tracing molecules are being evaluated for their efficacy in imaging cancer patients (NCT04285996).

Although the inhibition of integrin has not yet been successfully translated into a clinical trial for leukemia, integrins remain a valid target for cancer therapy, as they can serve as a targetable biomarker. Targeting the active form of the integrin β_7_ subunit, specifically the MMG49 epitope in the N-terminal region of active β_7_, showed multiple anti-myeloma effects *in vivo* without damaging normal hematopoietic cells (237). The efficacy of CAR T cells against α_v_β_3_ in melanoma and α_v_β_6_ in ovarian, breast, and pancreatic xenograft mice models has also been evaluated (238, 239) for integrin targeting CAR T therapy in hematological malignancies.

In summary, integrin blockades must be further investigated using preclinical systems that can accurately recapitulate the biological environments in patients, thus allowing the integrin blockade to exert anti-tumor effects (241) (**Table 4**). To overcome this challenge, integrins can serve as a good target for tumor imaging in patients or for immunotherapy, including CAR T therapy. As discussed in this section, integrin blockades have been shown to increase chemosensitivity of leukemia cells and provide support for further studies of integrins as a viable target to abolish CAM-DR in leukemia.

**Table 4:** Description of integrin inhibitors.

Drug name	Description	Indication	References
Natalizumab (Tysabri^®^)	Monoclonal antibody against α_4_	Multiple sclerosis and Crohn’s disease	(175)
Vedolizumab (Entivio)	Monoclonal antibody against α_4_β_7_	severe ulcerative colitis or Crohn’s disease	(242, 243)
Volociximab	Monoclonal antibody against integrin α_5_β_1_	Solid tumors including kidney, lung, ovarian cancer, melanoma, and pancreatic cancer	(244–247)
ATN-161	non-RGD based peptide targeting α_5_β_1_ and α_ν_β_3_	Solid tumors including prostate, colon, and hepatocellular cancers	(248–250)
Intetumumab (CNTO95)	human α_ν_ monoclonal antibody	Inhibition of tumor growth	(236, 251)
Etaracizumab (MEDI-522)	Monoclonal antibody against α_ν_β_3_	Psoriasis, kidney cancer	(252, 253)
Abituzumab (EMD525797)	Monoclonal antibody against α_ν_β_6_	Metastatic prostate cancer	(254, 255)
Cilengitide (EMD 121974)	first anti-angiogenic small molecule targeting the integrins α_v_β_3_, α_v_β_5_, and α_5_β_1_	Inhibition of endothelial cell–cell and cell–ECM interactions and angiogenesis	(256–259)
GLPG0187	a small molecule inhibitor for α_ν_β_1_, α_ν_β_3_, α_ν_β_5_, α_ν_β_6_, and α_5_β_1_	Solid tumors including high-grade gliomas and colorectal carcinoma	(260–262)
OS2966	Humanized monoclonal antibody against β_1_ integrins	Glioblastoma, meningioma, ALL, and AML	(236, 263)

## Conclusion

**Figure 5:**
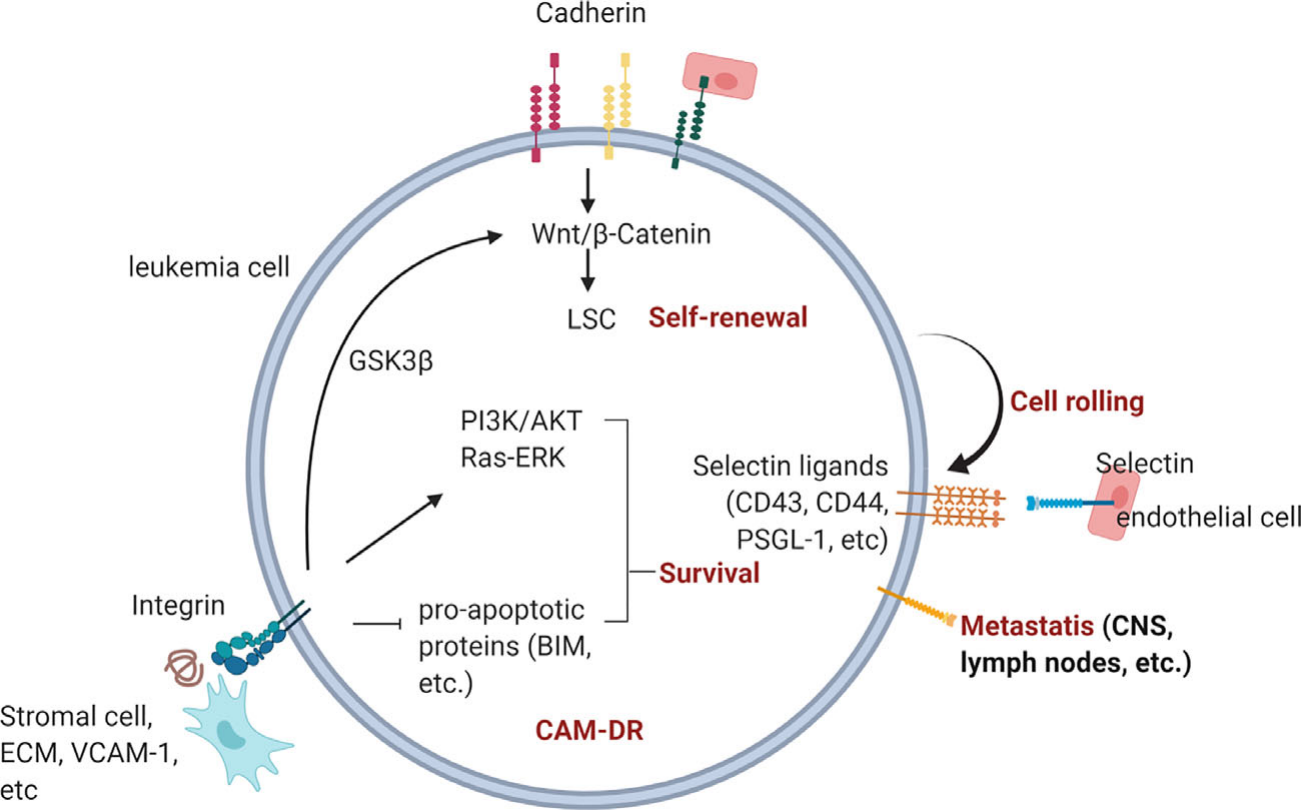
Overview of CAM-DR in leukemia. Cadherin, selectin, and integrin contribute to drug-resistance and metastasis upon engagement with their ligands in the BM. Homotropic engagment of cadherins can protect leukemia cells from chemotherapy (reference 92, 95) by modulating Wnt signaling and promote self-renewal of LSCs (reference 96, 97, 99). Leukemia cells can also bind to E-selectin expressed on endothelial cells through expressed selectin ligands on their surface (ref 132, 133). Interruption of E-selectin mediated interaction between leukemia and endothelial cells is actively being investigated in many clinical trials (ref 20). Integrin binding to BM stromal cells, ECM or counter receptors activates pro-survival signaling pathways such as PI3K/AKT and Ras/ERK pathway (ref 164, 221, 223, 229).

Despite advances in cancer therapy and the increased overall survival rate of cancer patients, the eradication of leukemia still remains a challenge. Pediatric ALL has a good prognosis overall, yet relapse and refractory disease remain a problem. AML has a worse prognosis, and there is an unmet need for the improvement of patient outcomes. The impact of the microenvironment on cancer cell progression and drug resistance has been often neglected, yet it is apparent that leukemia cells actively communicate and interact with the surrounding microenvironment for their survival. Therefore, disrupting the interactions between leukemia cells and the surrounding cells or ECM protein may lead to apoptosis or sensitization toward chemotherapy. Cadherins, selectins, and integrins are known cell adhesion molecules that are involved in CAM-DR in leukemia (**Figure 5**). Their aberrant expression and association in CAM-DR in different types of leukemia have been studied. Furthermore, a preclinical evaluation of the efficacy of the CAM blockade was performed on subtypes of leukemia and showed promising results with an anti-leukemic effect. The FDA has granted a Breakthrough Therapy designation and Fast Track designation for the E-selectin inhibitor Uproleselan, which shows both the urgency of finding an effective drug for leukemia treatment and the importance of microenvironment–leukemia interactions in leukemia treatment. The translation of more CAM inhibitors into a clinical platform will advance leukemia therapy and eradication of the disease.

## Author Contributions

HK and YK conceptualized the study. HK wrote and prepared the original draft. HK, YR, HO, and YK wrote, reviewed, and editing. YK Supervised the study and acquired the funding. All authors contributed to the article and approved the submitted version.

## Funding

This research was funded by NIH NCI, grant number CA172896 and the Alex’s Lemonade Stand Foundation (Y.K) Cure4Cam.

## Conflict of Interest

The authors declare that the research was conducted in the absence of any commercial or financial relationships that could be construed as a potential conflict of interest.

## References

[1] Gaynon PS Qu RP Chappell RJ Willoughby ML Tubergen DG Steinherz PG Survival after relapse in childhood acute lymphoblastic leukemia: impact of site and time to first relapse–the Children’s Cancer Group Experience. Cancer (1998) 82:1387–95. 10.1002/(SICI)1097-0142(19980401)82:7<1387::AID-CNCR24>3.0.CO;2-1 9529033

[2] Agarwal P Isringhausen S Li H Paterson AJ He J Gomariz A Mesenchymal Niche-Specific Expression of Cxcl12 Controls Quiescence of Treatment-Resistant Leukemia Stem Cells. Cell Stem Cell (2019) 24:769–84 e6. 10.1016/j.stem.2019.02.018 30905620 PMC6499704

[3] Zhang B Nguyen LXT Li L Zhao D Kumar B Wu H Bone marrow niche trafficking of miR-126 controls the self-renewal of leukemia stem cells in chronic myelogenous leukemia. Nat Med (2018) 24:450–62. 10.1038/nm.4499 PMC5965294 29505034

[4] Kunisaki Y Bruns I Scheiermann C Ahmed J Pinho S Zhang D Arteriolar niches maintain haematopoietic stem cell quiescence. Nature (2013) 502:637–43. 10.1038/nature12612 PMC3821873 24107994

[5] Akinduro O Weber TS Ang H Haltalli MLR Ruivo N Duarte D Proliferation dynamics of acute myeloid leukaemia and haematopoietic progenitors competing for bone marrow space. Nat Commun (2018) 9:519. 10.1038/s41467-017-02376-5 29410432 PMC5802720

[6] Matsunaga T Takemoto N Sato T Takimoto R Tanaka I Fujimi A Interaction between leukemic-cell VLA-4 and stromal fibronectin is a decisive factor for minimal residual disease of acute myelogenous leukemia. Nat Med (2003) 9:1158–65. 10.1038/nm909 12897778

[7] Walkley CR Olsen GH Dworkin S Fabb SA Swann J McArthur GA A microenvironment-induced myeloproliferative syndrome caused by retinoic acid receptor gamma deficiency. Cell (2007) 129:1097–110. 10.1016/j.cell.2007.05.014 PMC1974882 17574023

[8] Borowitz MJ Wood BL Devidas M Loh ML Raetz EA Salzer WL Prognostic significance of minimal residual disease in high risk B-ALL: a report from Children’s Oncology Group study AALL0232. Blood (2015) 126:964–71. 10.1182/blood-2015-03-633685 PMC4543229 26124497

[9] Campana D Role of minimal residual disease monitoring in adult and pediatric acute lymphoblastic leukemia. Hematol Oncol Clin North Am (2009) 23:1083–98, vii. 10.1016/j.hoc.2009.07.010 19825454 PMC2762949

[10] DiGiuseppe JA Fuller SG Borowitz MJ Overexpression of CD49f in precursor B-cell acute lymphoblastic leukemia: potential usefulness in minimal residual disease detection. Cytometry B Clin Cytom (2009) 76:150–5. 10.1002/cyto.b.20440 18831072

[11] Shalapour S Hof J Kirschner-Schwabe R Bastian L Eckert C Prada J High VLA-4 expression is associated with adverse outcome and distinct gene expression changes in childhood B-cell precursor acute lymphoblastic leukemia at first relapse. Haematologica (2011) 96:1627–35. 10.3324/haematol.2011.047993 PMC3208680 21828124

[12] Hunger SP Mullighan CG Acute Lymphoblastic Leukemia in Children. N Engl J Med (2015) 373:1541–52. 10.1056/NEJMra1400972 26465987

[13] Paul S Kantarjian H Jabbour EJ Adult Acute Lymphoblastic Leukemia. Mayo Clin Proc (2016) 91:1645–66. 10.1016/j.mayocp.2016.09.010 27814839

[14] Chiaretti S Messina M Foa R BCR/ABL1-like acute lymphoblastic leukemia: How to diagnose and treat? Cancer (2019) 125:194–204. 10.1002/cncr.31848 30561755

[15] De Kouchkovsky I Abdul-Hay M ‘Acute myeloid leukemia: a comprehensive review and 2016 update’. Blood Cancer J (2016) 6:e441. 10.1038/bcj.2016.50 27367478 PMC5030376

[16] Grimwade D Ivey A Huntly BJ Molecular landscape of acute myeloid leukemia in younger adults and its clinical relevance. Blood (2016) 127:29–41. 10.1182/blood-2015-07-604496 26660431 PMC4705608

[17] Rai KR Jain P Chronic lymphocytic leukemia (CLL)-Then and now. Am J Hematol (2016) 91:330–40. 10.1002/ajh.24282 26690614

[18] Sharma S Rai KR Chronic lymphocytic leukemia (CLL) treatment: So many choices, such great options. Cancer (2019) 125:1432–40. 10.1002/cncr.31931 30807655

[19] Houshmand M Simonetti G Circosta P Gaidano V Cignetti A Martinelli G Chronic myeloid leukemia stem cells. Leukemia (2019) 33:1543–56. 10.1038/s41375-019-0490-0 PMC6755964 31127148

[20] Radich J Yeung C Wu D New approaches to molecular monitoring in CML (and other diseases). Blood (2019) 134:1578–84. 10.1182/blood.2019000838 PMC9635586 31533919

[21] Duarte D Hawkins ED Lo Celso C The interplay of leukemia cells and the bone marrow microenvironment. Blood (2018) 131:1507–11. 10.1182/blood-2017-12-784132 29487069

[22] Vetrie D Helgason GV Copland M The leukaemia stem cell: similarities, differences and clinical prospects in CML and AML. Nat Rev Cancer (2020) 20:158–73. 10.1038/s41568-019-0230-9 31907378

[23] Arrigoni E Del Re M Galimberti S Restante G Rofi E Crucitta S Concise Review: Chronic Myeloid Leukemia: Stem Cell Niche and Response to Pharmacologic Treatment. Stem Cells Transl Med (2018) 7:305–14. 10.1002/sctm.17-0175 PMC5827745 29418079

[24] Villatoro A Konieczny J Cuminetti V Arranz L Leukemia Stem Cell Release From the Stem Cell Niche to Treat Acute Myeloid Leukemia. Front Cell Dev Biol (2020) 8:607. 10.3389/fcell.2020.00607 32754595 PMC7367216

[25] Qiu S Jia Y Xing H Yu T Yu J Yu P N-Cadherin and Tie2 positive CD34(+)CD38(-)CD123(+) leukemic stem cell populations can develop acute myeloid leukemia more effectively in NOD/SCID mice. Leuk Res (2014) 38:632–7. 10.1016/j.leukres.2014.03.007 24703771

[26] Zhi L Gao Y Yu C Zhang Y Zhang B Yang J N-Cadherin Aided in Maintaining the Characteristics of Leukemic Stem Cells. Anat Rec (Hoboken) (2016) 299:990–8. 10.1002/ar.23345 27064800

[27] Nishioka C Ikezoe T Pan B Xu K Yokoyama A MicroRNA-9 plays a role in interleukin-10-mediated expression of E-cadherin in acute myelogenous leukemia cells. Cancer Sci (2017) 108:685–95. 10.1111/cas.13170 PMC5406602 28107581

[28] Barbier V Erbani J Fiveash C Davies JM Tay J Tallack MR Endothelial E-selectin inhibition improves acute myeloid leukaemia therapy by disrupting vascular niche-mediated chemoresistance. Nat Commun (2020) 11:2042. 10.1038/s41467-020-15817-5 32341362 PMC7184728

[29] Krenn PW Koschmieder S Fassler R Kindlin-3 loss curbs chronic myeloid leukemia in mice by mobilizing leukemic stem cells from protective bone marrow niches. Proc Natl Acad Sci USA (2020) 117:24326–35. 10.1073/pnas.2009078117 PMC7533676 32929022

[30] Kuzelova K Obr A Markova J Gasova Z Integrin expression and adhesivity to fibronectin in primary acute myeloid leukemia cells: Impact of NPM1 and FLT3 mutations. Eur J Haematol (2020) 105:578–87. 10.1111/ejh.13488 32668024

[31] Asada N Kunisaki Y Pierce H Wang Z Fernandez NF Birbrair A Differential cytokine contributions of perivascular haematopoietic stem cell niches. Nat Cell Biol (2017) 19:214–23. 10.1038/ncb3475 PMC5467892 28218906

[32] Sugiyama T Kohara H Noda M Nagasawa T Maintenance of the hematopoietic stem cell pool by CXCL12-CXCR4 chemokine signaling in bone marrow stromal cell niches. Immunity (2006) 25:977–88. 10.1016/j.immuni.2006.10.016 17174120

[33] Acar M Kocherlakota KS Murphy MM Peyer JG Oguro H Inra CN Deep imaging of bone marrow shows non-dividing stem cells are mainly perisinusoidal. Nature (2015) 526:126–30. 10.1038/nature15250 PMC4850557 26416744

[34] Calvi LM Adams GB Weibrecht KW Weber JM Olson DP Knight MC Osteoblastic cells regulate the haematopoietic stem cell niche. Nature (2003) 425:841–6. 10.1038/nature02040 14574413

[35] Naveiras O Nardi V Wenzel PL Hauschka PV Fahey F Daley GQ Bone-marrow adipocytes as negative regulators of the haematopoietic microenvironment. Nature (2009) 460:259–63. 10.1038/nature08099 PMC2831539 19516257

[36] Mendez-Ferrer S Lucas D Battista M Frenette PS Haematopoietic stem cell release is regulated by circadian oscillations. Nature (2008) 452:442–7. 10.1038/nature06685 18256599

[37] Yamazaki S Ema H Karlsson G Yamaguchi T Miyoshi H Shioda S Nonmyelinating Schwann cells maintain hematopoietic stem cell hibernation in the bone marrow niche. Cell (2011) 147:1146–58. 10.1016/j.cell.2011.09.053 22118468

[38] Bruns I Lucas D Pinho S Ahmed J Lambert MP Kunisaki Y Megakaryocytes regulate hematopoietic stem cell quiescence through CXCL4 secretion. Nat Med (2014) 20:1315–20. 10.1038/nm.3707 PMC4258871 25326802

[39] Mukaida N Tanabe Y Baba T Chemokines as a Conductor of Bone Marrow Microenvironment in Chronic Myeloid Leukemia. Int J Mol Sci (2017) 18. 10.3390/ijms18081824 PMC5578209 28829353

[40] Kumar B Garcia M Weng L Jung X Murakami JL Hu X Acute myeloid leukemia transforms the bone marrow niche into a leukemia-permissive microenvironment through exosome secretion. Leukemia (2018) 32:575–87. 10.1038/leu.2017.259 PMC5843902 28816238

[41] Wang B Wang X Hou D Huang Q Zhan W Chen C Exosomes derived from acute myeloid leukemia cells promote chemoresistance by enhancing glycolysis-mediated vascular remodeling. J Cell Physiol (2020) 234:10602–14 10.1002/jcp.27735 30417360

[42] Salati S Salvestrini V Carretta C Genovese E Rontauroli S Zini R Deregulated expression of miR-29a-3p, miR-494-3p and miR-660-5p affects sensitivity to tyrosine kinase inhibitors in CML leukemic stem cells. Oncotarget (2017) 8:49451–69. 10.18632/oncotarget.17706 PMC5564781 28533480

[43] Sison EAR Kurre P Kim YM Understanding the bone marrow microenvironment in hematologic malignancies: A focus on chemokine, integrin, and extracellular vesicle signaling. Pediatr Hematol Oncol (2017) 34:365–78. 10.1080/08880018.2017.1395938 PMC6516746 29211600

[44] Reagan MR Rosen CJ Navigating the bone marrow niche: translational insights and cancer-driven dysfunction. Nat Rev Rheumatol (2016) 12:154–68. 10.1038/nrrheum.2015.160 PMC4947935 26607387

[45] Shin JW Mooney DJ Extracellular matrix stiffness causes systematic variations in proliferation and chemosensitivity in myeloid leukemias. Proc Natl Acad Sci USA (2016) 113:12126–31. 10.1073/pnas.1611338113 PMC5086998 27790998

[46] Usmani S Sivagnanalingam U Tkachenko O Nunez L Shand JC Mullen CA Support of acute lymphoblastic leukemia cells by nonmalignant bone marrow stromal cells. Oncol Lett (2019) 17:5039–49. 10.3892/ol.2019.10188 PMC6507394 31186715

[47] Garrido SM Appelbaum FR Willman CL Banker DE Acute myeloid leukemia cells are protected from spontaneous and drug-induced apoptosis by direct contact with a human bone marrow stromal cell line (HS-5). Exp Hematol (2001) 29:448–57. 10.1016/S0301-472X(01)00612-9 11301185

[48] Moses BS Slone WL Thomas P Evans R Piktel D Angel PM Bone marrow microenvironment modulation of acute lymphoblastic leukemia phenotype. Exp Hematol (2016) 44:50–9 e1-2. 10.1016/j.exphem.2015.09.003 26407636 PMC4684957

[49] Mohle R Bautz F Rafii S Moore MA Brugger W Kanz L The chemokine receptor CXCR-4 is expressed on CD34+ hematopoietic progenitors and leukemic cells and mediates transendothelial migration induced by stromal cell-derived factor-1. Blood (1998) 91:4523–30. 10.1182/blood.V91.12.4523.412k04_4523_4530 9616148

[50] Levesque J-P Winkler IG Cell Adhesion Molecules in Normal and Malignant Hematopoiesis: from Bench to Bedside. Curr Stem Cell Rep (2016) 2:356–67. 10.1007/s40778-016-0066-0

[51] Windisch R Pirschtat N Kellner C Chen-Wichmann L Lausen J Humpe A Oncogenic Deregulation of Cell Adhesion Molecules in Leukemia. Cancers (Basel) (2019) 11. 10.3390/cancers11030311 PMC6468598 30841639

[52] Gruszka AM Valli D Restelli C Alcalay M Adhesion Deregulation in Acute Myeloid Leukaemia. Cells (2019) 8. 10.3390/cells8010066 PMC6356639 30658474

[53] Frenette PS Wagner DD Adhesion molecules–Part 1. N Engl J Med (1996) 334:1526–9. 10.1056/NEJM199606063342308 8618609

[54] Hirano S Nose A Hatta K Kawakami A Takeichi M Calcium-dependent cell-cell adhesion molecules (cadherins): subclass specificities and possible involvement of actin bundles. J Cell Biol (1987) 105:2501–10. 10.1083/jcb.105.6.2501 PMC2114731 3320048

[55] Cailliez F Lavery R Cadherin mechanics and complexation: the importance of calcium binding. Biophys J (2005) 89:3895–903. 10.1529/biophysj.105.067322 PMC1366956 16183887

[56] Kim SA Tai CY Mok LP Mosser EA Schuman EM Calcium-dependent dynamics of cadherin interactions at cell-cell junctions. Proc Natl Acad Sci USA (2011) 108:9857–62. 10.1073/pnas.1019003108 PMC3116393 21613566

[57] McEver RP Selectins: initiators of leucocyte adhesion and signalling at the vascular wall. Cardiovasc Res (2015) 107:331–9. 10.1093/cvr/cvv154 PMC4592324 25994174

[58] Zhang K Chen J The regulation of integrin function by divalent cations. Cell Adh Migr (2012) 6:20–9. 10.4161/cam.18702 PMC3364134 22647937

[59] Tiwari S Askari JA Humphries MJ Bulleid NJ Divalent cations regulate the folding and activation status of integrins during their intracellular trafficking. J Cell Sci (2011) 124:1672–80. 10.1242/jcs.084483 PMC3085436 21511727

[60] Calderwood DA Integrin activation. J Cell Sci (2004) 117:657–66. 10.1242/jcs.01014 14754902

[61] Galan-Diez M Cuesta-Dominguez A Kousteni S The Bone Marrow Microenvironment in Health and Myeloid Malignancy. Cold Spring Harb Perspect Med (2018) 8. 10.1101/cshperspect.a031328 PMC6027930 28963115

[62] Zhao M Tao F Venkatraman A Li Z Smith SE Unruh J N-Cadherin-Expressing Bone and Marrow Stromal Progenitor Cells Maintain Reserve Hematopoietic Stem Cells. Cell Rep (2019) 26:652–69.e6. 10.1016/j.celrep.2018.12.093 30650358 PMC6890378

[63] van Buul JD Voermans C van den Berg V Anthony EC Mul FP van Wetering S Migration of human hematopoietic progenitor cells across bone marrow endothelium is regulated by vascular endothelial cadherin. J Immunol (2002) 168:588–96. 10.4049/jimmunol.168.2.588 11777950

[64] Miyake K Medina K Ishihara K Kimoto M Auerbach R Kincade PW A VCAM-like adhesion molecule on murine bone marrow stromal cells mediates binding of lymphocyte precursors in culture. J Cell Biol (1991) 114:557–65. 10.1083/jcb.114.3.557 PMC2289098 1713592

[65] Schweitzer KM Drager AM van der Valk P Thijsen SF Zevenbergen A Theijsmeijer AP Constitutive expression of E-selectin and vascular cell adhesion molecule-1 on endothelial cells of hematopoietic tissues. Am J Pathol (1996) 148:165–75. PMC1861610 8546203

[66] Xia YF Liu LP Zhong CP Geng JG NF-kappaB activation for constitutive expression of VCAM-1 and ICAM-1 on B lymphocytes and plasma cells. Biochem Biophys Res Commun (2001) 289:851–6. 10.1006/bbrc.2001.6067 11735124

[67] Malara A Currao M Gruppi C Celesti G Viarengo G Buracchi C Megakaryocytes contribute to the bone marrow-matrix environment by expressing fibronectin, type IV collagen, and laminin. Stem Cells (2014) 32:926–37. 10.1002/stem.1626 PMC4096110 24357118

[68] Ulyanova T Scott LM Priestley GV Jiang Y Nakamoto B Koni PA VCAM-1 expression in adult hematopoietic and nonhematopoietic cells is controlled by tissue-inductive signals and reflects their developmental origin. Blood (2005) 106:86–94. 10.1182/blood-2004-09-3417 15769895 PMC1895134

[69] Zhang P Zhang C Li J Han J Liu X Yang H The physical microenvironment of hematopoietic stem cells and its emerging roles in engineering applications. Stem Cell Res Ther (2019) 10:327. 10.1186/s13287-019-1422-7 31744536 PMC6862744

[70] Nilsson SK Debatis ME Dooner MS Madri JA Quesenberry PJ Becker PS Immunofluorescence characterization of key extracellular matrix proteins in murine bone marrow in situ. J Histochem Cytochem (1998) 46:371–7. 10.1177/002215549804600311 9487119

[71] Tikhonova AN Dolgalev I Hu H Sivaraj KK Hoxha E Cuesta-Dominguez A The bone marrow microenvironment at single-cell resolution. Nature (2019) 569:222–8. 10.1038/s41586-019-1104-8 PMC6607432 30971824

[72] Kokkaliaris KD Scadden DT Cell interactions in the bone marrow microenvironment affecting myeloid malignancies. Blood Adv (2020) 4:3795–803. 10.1182/bloodadvances.2020002127 PMC7422101 32780848

[73] Kumar R Godavarthy PS Krause DS The bone marrow microenvironment in health and disease at a glance. J Cell Sci (2018) 131. 10.1242/jcs.201707 29472498

[74] Ciciarello M Corradi G Loscocco F Visani G Monaco F Cavo M The Yin and Yang of the Bone Marrow Microenvironment: Pros and Cons of Mesenchymal Stromal Cells in Acute Myeloid Leukemia. Front Oncol (2019) 9:1135. 10.3389/fonc.2019.01135 31709192 PMC6823864

[75] Wang J Liu X Qiu Y Shi Y Cai J Wang B Cell adhesion-mediated mitochondria transfer contributes to mesenchymal stem cell-induced chemoresistance on T cell acute lymphoblastic leukemia cells. J Hematol Oncol (2018) 11:11. 10.1186/s13045-018-0554-z 29357914 PMC5778754

[76] Ivanov DB Philippova MP Tkachuk VA Structure and functions of classical cadherins. Biochem (Mosc) (2001) 66:1174–86. 10.1023/A:1012445316415 11736639

[77] Saito M Tucker DK Kohlhorst D Niessen CM Kowalczyk AP Classical and desmosomal cadherins at a glance. J Cell Sci (2012) 125:2547–52. 10.1242/jcs.066654 PMC3403229 22833291

[78] Hayashi S Takeichi M Emerging roles of protocadherins: from self-avoidance to enhancement of motility. J Cell Sci (2015) 128:1455–64. 10.1242/jcs.166306 25749861

[79] Tsukasaki Y Miyazaki N Matsumoto A Nagae S Yonemura S Tanoue T Giant cadherins Fat and Dachsous self-bend to organize properly spaced intercellular junctions. Proc Natl Acad Sci USA (2014) 111:16011–6. 10.1073/pnas.1418990111 PMC4234546 25355906

[80] Yu W Yang L Li T Zhang Y Cadherin Signaling in Cancer: Its Functions and Role as a Therapeutic Target. Front Oncol (2019) 9:989. 10.3389/fonc.2019.00989 31637214 PMC6788064

[81] van Roy F Beyond E-cadherin: roles of other cadherin superfamily members in cancer. Nat Rev Cancer (2014) 14:121–34. 10.1038/nrc3647 24442140

[82] Vieira AF Paredes J P-cadherin and the journey to cancer metastasis. Mol Cancer (2015) 14:178. 10.1186/s12943-015-0448-4 26438065 PMC4595126

[83] Rimm DL Koslov ER Kebriaei P Cianci CD Morrow JS Alpha 1(E)-catenin is an actin-binding and -bundling protein mediating the attachment of F-actin to the membrane adhesion complex. Proc Natl Acad Sci USA (1995) 92:8813–7. 10.1073/pnas.92.19.8813 PMC41057 7568023

[84] Drees F Pokutta S Yamada S Nelson WJ Weis WI Alpha-catenin is a molecular switch that binds E-cadherin-beta-catenin and regulates actin-filament assembly. Cell (2005) 123:903–15. 10.1016/j.cell.2005.09.021 PMC3369825 16325583

[85] Shapiro L Weis WI Structure and biochemistry of cadherins and catenins. Cold Spring Harb Perspect Biol (2009) 1:a003053. 10.1101/cshperspect.a003053 20066110 PMC2773639

[86] Kourtidis A Lu R Pence LJ Anastasiadis PZ A central role for cadherin signaling in cancer. Exp Cell Res (2017) 358:78–85. 10.1016/j.yexcr.2017.04.006 28412244 PMC5544584

[87] Gottardi CJ Wong E Gumbiner BM E-cadherin suppresses cellular transformation by inhibiting beta-catenin signaling in an adhesion-independent manner. J Cell Biol (2001) 153:1049–60. 10.1083/jcb.153.5.1049 PMC2174337 11381089

[88] Wheelock MJ Shintani Y Maeda M Fukumoto Y Johnson KR Cadherin switching. J Cell Sci (2008) 121:727–35. 10.1242/jcs.000455 18322269

[89] Gheldof A Berx G Cadherins and epithelial-to-mesenchymal transition. Prog Mol Biol Transl Sci (2013) 116:317–36. 10.1016/B978-0-12-394311-8.00014-5 23481201

[90] Melki JR Vincent PC Brown RD Clark SJ Hypermethylation of E-cadherin in leukemia. Blood (2000) 95:3208–13. 10.1182/blood.V95.10.3208.010k02_3208_3213 10807790

[91] Corn PG Smith BD Ruckdeschel ES Douglas D Baylin SB Herman JG E-cadherin expression is silenced by 5’ CpG island methylation in acute leukemia. Clin Cancer Res (2000) 6:4243–8. 11106238

[92] Zhang B Li M McDonald T Holyoake TL Moon RT Campana D Microenvironmental protection of CML stem and progenitor cells from tyrosine kinase inhibitors through N-cadherin and Wnt-beta-catenin signaling. Blood (2013) 121:1824–38. 10.1182/blood-2012-02-412890 PMC3591802 23299311

[93] Zhang B Groffen J Heisterkamp N Increased resistance to a farnesyltransferase inhibitor by N-cadherin expression in Bcr/Abl-P190 lymphoblastic leukemia cells. Leukemia (2007) 21:1189–97. 10.1038/sj.leu.2404667 17392819

[94] Akers SM O’Leary HA Minnear FL Craig MD Vos JA Coad JE VE-cadherin and PECAM-1 enhance ALL migration across brain microvascular endothelial cell monolayers. Exp Hematol (2010) 38:733–43. 10.1016/j.exphem.2010.05.001 PMC2929642 20470859

[95] Chen C Zhang HX Wang M Song XG Cao J Wang L Stromal cells attenuate the cytotoxicity of imatinib on Philadelphia chromosome-positive leukemia cells by up-regulating the VE-cadherin/beta-catenin signal. Leuk Res (2014) 38:1460–8. 10.1016/j.leukres.2014.09.012 25443888

[96] Howard S Deroo T Fujita Y Itasaki N A positive role of cadherin in Wnt/beta-catenin signalling during epithelial-mesenchymal transition. PLoS One (2011) 6:e23899. 10.1371/journal.pone.0023899 21909376 PMC3166074

[97] Wang L O’Leary H Fortney J Gibson LF Ph+/VE-cadherin+ identifies a stem cell like population of acute lymphoblastic leukemia sustained by bone marrow niche cells. Blood (2007) 110:3334–44. 10.1182/blood-2007-01-068122 PMC2200915 17638851

[98] O’Leary H Akers SM Piktel D Walton C Fortney JE Martin KH VE-cadherin Regulates Philadelphia Chromosome Positive Acute Lymphoblastic Leukemia Sensitivity to Apoptosis. Cancer Microenviron (2010) 3:67–81. 10.1007/s12307-010-0035-6 21209775 PMC2990486

[99] Zhi L Wang M Rao Q Yu F Mi Y Wang J Enrichment of N-Cadherin and Tie2-bearing CD34+/CD38-/CD123+ leukemic stem cells by chemotherapy-resistance. Cancer Lett (2010) 296:65–73. 10.1016/j.canlet.2010.03.021 20444543

[100] Kuhn K Cott C Bohler S Aigal S Zheng S Villringer S The interplay of autophagy and beta-Catenin signaling regulates differentiation in acute myeloid leukemia. Cell Death Discovery (2015) 1:15031. 10.1038/cddiscovery.2015.31 27551462 PMC4979480

[101] Liang SM Lu YJ Ko BS Jan YJ Shyue SK Yet SF Cordycepin disrupts leukemia association with mesenchymal stromal cells and eliminates leukemia stem cell activity. Sci Rep (2017) 7:43930. 10.1038/srep43930 28266575 PMC5339716

[102] Bosse RC Wasserstrom B Meacham A Wise E Drusbosky L Walter GA Chemosensitizing AML cells by targeting bone marrow endothelial cells. Exp Hematol (2016) 44:363–77.e5. 10.1016/j.exphem.2016.02.003 26898708

[103] Yarom N Stewart D Avruch L Malik R Wells J Jonker DJ ADH-1 in the treatment of metastatic adrenocortical carcinoma–case report. Anticancer Res (2011) 31:3921–5. 22110220

[104] Yarom N Stewart D Malik R Wells J Avruch L Jonker DJ Phase I clinical trial of Exherin (ADH-1) in patients with advanced solid tumors. Curr Clin Pharmacol (2013) 8:81–8. 10.2174/157488413804810576 22280327

[105] Perotti A Sessa C Mancuso A Noberasco C Cresta S Locatelli A Clinical and pharmacological phase I evaluation of Exherin (ADH-1), a selective anti-N-cadherin peptide in patients with N-cadherin-expressing solid tumours. Ann Oncol (2009) 20:741–5. 10.1093/annonc/mdn695 19190075

[106] Beasley GM McMahon N Sanders G Augustine CK Selim MA Peterson B A phase 1 study of systemic ADH-1 in combination with melphalan via isolated limb infusion in patients with locally advanced in-transit malignant melanoma. Cancer (2009) 115:4766–74. 10.1002/cncr.24509 PMC7533793 19637344

[107] Beasley GM Riboh JC Augustine CK Zager JS Hochwald SN Grobmyer SR Prospective multicenter phase II trial of systemic ADH-1 in combination with melphalan via isolated limb infusion in patients with advanced extremity melanoma. J Clin Oncol (2011) 29:1210–5. 10.1200/JCO.2010.32.1224 PMC4668281 21343562

[108] Mrozik KM Blaschuk OW Cheong CM Zannettino ACW Vandyke K N-cadherin in cancer metastasis, its emerging role in haematological malignancies and potential as a therapeutic target in cancer. BMC Cancer (2018) 18:939. 10.1186/s12885-018-4845-0 30285678 PMC6167798

[109] Atar D Petzelbauer P Schwitter J Huber K Rensing B Kasprzak JD Effect of intravenous FX06 as an adjunct to primary percutaneous coronary intervention for acute ST-segment elevation myocardial infarction results of the F.I.R.E. (Efficacy of FX06 in the Prevention of Myocardial Reperfusion Injury) trial. J Am Coll Cardiol (2009) 53:720–9. 10.1016/j.jacc.2008.12.017 19232907

[110] Roesner JP Petzelbauer P Koch A Tran N Iber T Vagts DA Bbeta15-42 (FX06) reduces pulmonary, myocardial, liver, and small intestine damage in a pig model of hemorrhagic shock and reperfusion. Crit Care Med (2009) 37:598–605. 10.1097/CCM.0b013e3181959a12 19114899

[111] Wang YP Wang QY Li CH Li XW COX-2 inhibition by celecoxib in epithelial ovarian cancer attenuates E-cadherin suppression through reduced Snail nuclear translocation. Chem Biol Interact (2018) 292:24–9. 10.1016/j.cbi.2018.06.020 29932878

[112] Zhou Y Ran J Tang C Wu J Honghua L Xingwen L Effect of celecoxib on E-cadherin, VEGF, Microvessel density and apoptosis in gastric cancer. Cancer Biol Ther (2007) 6:269–75. 10.4161/cbt.6.2.3629 17224647

[113] Chen Z Liu M Liu X Huang S Li L Song B COX-2 regulates E-cadherin expression through the NF-kappaB/Snail signaling pathway in gastric cancer. Int J Mol Med (2013) 32:93–100. 10.3892/ijmm.2013.1376 23670240

[114] Wiedemann D Schneeberger S Friedl P Zacharowski K Wick N Boesch F The fibrin-derived peptide Bbeta(15-42) significantly attenuates ischemia-reperfusion injury in a cardiac transplant model. Transplantation (2010) 89:824–9. 10.1097/TP.0b013e3181ccd822 20405575

[115] Bowler MA Raddatz MA Johnson CL Lindman BR Merryman WD Celecoxib Is Associated With Dystrophic Calcification and Aortic Valve Stenosis. JACC Basic Transl Sci (2019) 4:135–43. 10.1016/j.jacbts.2018.12.003 PMC6488810 31061914

[116] Assefnia S Dakshanamurthy S Guidry Auvil JM Hampel C Anastasiadis PZ Kallakury B Cadherin-11 in poor prognosis malignancies and rheumatoid arthritis: common target, common therapies. Oncotarget (2014) 5:1458–74. 10.18632/oncotarget.1538 PMC4039224 24681547

[117] Liu B Yan S Qu L Zhu J Celecoxib enhances anticancer effect of cisplatin and induces anoikis in osteosarcoma via PI3K/Akt pathway. Cancer Cell Int (2017) 17:1. 10.1186/s12935-016-0378-2 28053596 PMC5209942

[118] Liu R Zheng J Li C Pang Y Zheng Q Xu X Celecoxib induces epithelial-mesenchymal transition in epithelial ovarian cancer cells via regulating ZEB1 expression. Arch Gynecol Obstet (2015) 291:1361–9. 10.1007/s00404-014-3555-3 25424898

[119] Robison NJ Campigotto F Chi SN Manley PE Turner CD Zimmerman MA A phase II trial of a multi-agent oral antiangiogenic (metronomic) regimen in children with recurrent or progressive cancer. Pediatr Blood Cancer (2014) 61:636–42. 10.1002/pbc.24794 PMC4285784 24123865

[120] Riva B De Dominici M Gnemmi I Mariani SA Minassi A Minieri V Celecoxib inhibits proliferation and survival of chronic myelogeous leukemia (CML) cells via AMPK-dependent regulation of beta-catenin and mTORC1/2. Oncotarget (2016) 7:81555–70. 10.18632/oncotarget.13146 PMC5348412 27835591

[121] Lu Y Liu LL Liu SS Fang ZG Zou Y Deng XB Celecoxib suppresses autophagy and enhances cytotoxicity of imatinib in imatinib-resistant chronic myeloid leukemia cells. J Transl Med (2016) 14:270. 10.1186/s12967-016-1012-8 27645552 PMC5029099

[122] Lu Y Liu XF Liu TR Fan RF Xu YC Zhang XZ Celecoxib exerts antitumor effects in HL-60 acute leukemia cells and inhibits autophagy by affecting lysosome function. BioMed Pharmacother (2016) 84:1551–7. 10.1016/j.biopha.2016.11.026 27884749

[123] Ruan Y Kim HN Ogana H Kim YM Wnt Signaling in Leukemia and Its Bone Marrow Microenvironment. Int J Mol Sci (2020) 21. 10.3390/ijms21176247 PMC7503842 32872365

[124] Borsig L Selectins in cancer immunity. Glycobiology (2018) 28:648–55. 10.1093/glycob/cwx105 PMC6711759 29272415

[125] Patel KD Cuvelier SL Wiehler S Selectins: critical mediators of leukocyte recruitment. Semin Immunol (2002) 14:73–81. 10.1006/smim.2001.0344 11978079

[126] Cummings RD Smith DF The selectin family of carbohydrate-binding proteins: structure and importance of carbohydrate ligands for cell adhesion. Bioessays (1992) 14:849–56. 10.1002/bies.950141210 1285423

[127] Redondo-Munoz J Garcia-Pardo A Teixido J Molecular Players in Hematologic Tumor Cell Trafficking. Front Immunol (2019) 10:156. 10.3389/fimmu.2019.00156 30787933 PMC6372527

[128] Choudhary D Hegde P Voznesensky O Choudhary S Kopsiaftis S Claffey KP Increased expression of L-selectin (CD62L) in high-grade urothelial carcinoma: A potential marker for metastatic disease. Urol Oncol (2015) 33:387 e17–27. 10.1016/j.urolonc.2014.12.009 PMC4510033 25618296

[129] Esposito M Mondal N Greco TM Wei Y Spadazzi C Lin SC Bone vascular niche E-selectin induces mesenchymal-epithelial transition and Wnt activation in cancer cells to promote bone metastasis. Nat Cell Biol (2019) 21:627–39. 10.1038/s41556-019-0309-2 PMC6556210 30988423

[130] Ishikawa T Imura A Tanaka K Shirane H Okuma M Uchiyama T E-selectin and vascular cell adhesion molecule-1 mediate adult T-cell leukemia cell adhesion to endothelial cells. Blood (1993) 82:1590–8. 10.1182/blood.V82.5.1590.bloodjournal8251590 7689874

[131] Morita K Tokushige C Maeda S Kiyose H Noura M Iwai A RUNX transcription factors potentially control E-selectin expression in the bone marrow vascular niche in mice. Blood Adv (2018) 2:509–15. 10.1182/bloodadvances.2017009324 PMC5851413 29500219

[132] Bistrian R Dorn A Mobest DC Ruster B Ludwig R Scheele J Shear stress-mediated adhesion of acute myeloid leukemia and KG-1 cells to endothelial cells involves functional P-selectin. Stem Cells Dev (2009) 18:1235–42. 10.1089/scd.2008.0380 19105599

[133] Spertini C Baisse B Bellone M Gikic M Smirnova T Spertini O Acute Myeloid and Lymphoblastic Leukemia Cell Interactions with Endothelial Selectins: Critical Role of PSGL-1, CD44 and CD43. Cancers (Basel) (2019) 11. 10.3390/cancers11091253 PMC6770432 31461905

[134] Hadzijusufovic E Albrecht-Schgoer K Huber K Hoermann G Grebien F Eisenwort G Nilotinib-induced vasculopathy: identification of vascular endothelial cells as a primary target site. Leukemia (2017) 31:2388–97. 10.1038/leu.2017.245 PMC5669463 28757617

[135] Doroszko A Niedzielska E Jakubowski M Porwolik J Turek-Jakubowska A Szahidewicz-Krupska E Endothelial Function in Children with Acute Lymphoblastic Leukemia (ALL) May Reflect the Clinical Outcome. BioMed Res Int (2018) 2018:7918091. 10.1155/2018/7918091 30534565 PMC6252207

[136] Yu X Zhang H Yuan M Zhang P Wang Y Zheng M Identification and characterization of a murine model of BCRABL1+ acute Blymphoblastic leukemia with central nervous system metastasis. Oncol Rep (2019) 42:521–32. 10.3892/or.2019.7184 PMC6610040 31173268

[137] Festuccia C Mancini A Gravina GL Colapietro A Vetuschi A Pompili S Dual CXCR4 and E-Selectin Inhibitor, GMI-1359, Shows Anti-Bone Metastatic Effects and Synergizes with Docetaxel in Prostate Cancer Cell Intraosseous Growth. Cells (2019) 9. 10.3390/cells9010032 PMC7017374 31877673

[138] Muz B Azab F Fiala M King J Kohnen D Fogler WE Inhibition of E-Selectin (GMI-1271) or E-selectin together with CXCR4 (GMI-1359) re-sensitizes multiple myeloma to therapy. Blood Cancer J (2019) 9:68. 10.1038/s41408-019-0227-3 31431613 PMC6702213

[139] Ataga KI Kutlar A Kanter J Liles D Cancado R Friedrisch J Crizanlizumab for the Prevention of Pain Crises in Sickle Cell Disease. N Engl J Med (2017) 376:429–39. 10.1056/NEJMoa1611770 PMC5481200 27959701

[140] Davidson BP Kaufmann BA Belcik JT Xie A Qi Y Lindner JR Detection of antecedent myocardial ischemia with multiselectin molecular imaging. J Am Coll Cardiol (2012) 60:1690–7. 10.1016/j.jacc.2012.07.027 PMC3856914 23021335

[141] Cheadle C Watkins T Ehrlich E Barnes K Gaber AO Hemmerich S Effects of anti-adhesive therapy on kidney biomarkers of ischemia reperfusion injury in human deceased donor kidney allografts. Clin Transplant (2011) 25:766–75. 10.1111/j.1399-0012.2010.01365.x 21114535

[142] Natoni A Smith TAG Keane N McEllistrim C Connolly C Jha A E-selectin ligands recognised by HECA452 induce drug resistance in myeloma, which is overcome by the E-selectin antagonist, GMI-1271. Leukemia (2017) 31:2642–51. 10.1038/leu.2017.123 PMC5729350 28439107

[143] Gaber AO Mulgaonkar S Kahan BD Woodle ES Alloway R Bajjoka I YSPSL (rPSGL-Ig) for improvement of early renal allograft function: a double-blind, placebo-controlled, multi-center Phase IIa study. Clin Transplant (2011) 25:523–33. 10.1111/j.1399-0012.2010.01295.x 20573162

[144] Houshmand M Blanco TM Circosta P Yazdi N Kazemi A Saglio G Bone marrow microenvironment: The guardian of leukemia stem cells. World J Stem Cells (2019) 11:476–90. 10.4252/wjsc.v11.i8.476 PMC6716085 31523368

[145] Karantanou C Godavarthy PS Krause DS Targeting the bone marrow microenvironment in acute leukemia. Leuk Lymphoma (2018) 59:2535–45. 10.1080/10428194.2018.1434886 29431560

[146] Tabe Y Konopleva M Role of Microenvironment in Resistance to Therapy in AML. Curr Hematol Malig Rep (2015) 10:96–103. 10.1007/s11899-015-0253-6 25921386 PMC4447522

[147] Ruoslahti E RGD and other recognition sequences for integrins. Annu Rev Cell Dev Biol (1996) 12:697–715. 10.1146/annurev.cellbio.12.1.697 8970741

[148] Moreno-Layseca P Icha J Hamidi H Ivaska J Integrin trafficking in cells and tissues. Nat Cell Biol (2019) 21:122–32. 10.1038/s41556-018-0223-z PMC6597357 30602723

[149] Kechagia JZ Ivaska J Roca-Cusachs P Integrins as biomechanical sensors of the microenvironment. Nat Rev Mol Cell Biol (2019) 20:457–73. 10.1038/s41580-019-0134-2 31182865

[150] Calderwood DA Campbell ID Critchley DR Talins and kindlins: partners in integrin-mediated adhesion. Nat Rev Mol Cell Biol (2013) 14:503–17. 10.1038/nrm3624 PMC4116690 23860236

[151] Giancotti FG Ruoslahti E Integrin signaling. Science (1999) 285:1028–32. 10.1126/science.285.5430.1028 10446041

[152] Cooper J Giancotti FG Integrin Signaling in Cancer: Mechanotransduction, Stemness, Epithelial Plasticity, and Therapeutic Resistance. Cancer Cell (2019) 35:347–67. 10.1016/j.ccell.2019.01.007 PMC6684107 30889378

[153] Hamidi H Pietila M Ivaska J The complexity of integrins in cancer and new scopes for therapeutic targeting. Br J Cancer (2016) 115:1017–23. 10.1038/bjc.2016.312 PMC5117799 27685444

[154] Bridgewater RE Norman JC Caswell PT Integrin trafficking at a glance. J Cell Sci (2012) 125:3695–701. 10.1242/jcs.095810 PMC3462077 23027580

[155] Sun Z Guo SS Fassler R Integrin-mediated mechanotransduction. J Cell Biol (2016) 215:445–56. 10.1083/jcb.201609037 PMC5119943 27872252

[156] Pfaff M Gohring W Brown JC Timpl R Binding of purified collagen receptors (alpha 1 beta 1, alpha 2 beta 1) and RGD-dependent integrins to laminins and laminin fragments. Eur J Biochem (1994) 225:975–84. 10.1111/j.1432-1033.1994.0975b.x 7525287

[157] Miyake S Sakurai T Okumura K Yagita H Identification of collagen and laminin receptor integrins on murine T lymphocytes. Eur J Immunol (1994) 24:2000–5. 10.1002/eji.1830240910 7522156

[158] Hemler ME Jacobson JG Brenner MB Mann D Strominger JL VLA-1: a T cell surface antigen which defines a novel late stage of human T cell activation. Eur J Immunol (1985) 15:502–8. 10.1002/eji.1830150515 2986987

[159] Roberts AI Brolin RE Ebert EC Integrin alpha1beta1 (VLA-1) mediates adhesion of activated intraepithelial lymphocytes to collagen. Immunology (1999) 97:679–85. 10.1046/j.1365-2567.1999.00812.x PMC2326887 10457223

[160] Deschaseaux F Charbord P Human marrow stromal precursors are alpha 1 integrin subunit-positive. J Cell Physiol (2000) 184:319–25. 10.1002/1097-4652(200009)184:3<319::AID-JCP5>3.0.CO;2-N 10911362

[161] Gardner H Kreidberg J Koteliansky V Jaenisch R Deletion of integrin alpha 1 by homologous recombination permits normal murine development but gives rise to a specific deficit in cell adhesion. Dev Biol (1996) 175:301–13. 10.1006/dbio.1996.0116 8626034

[162] Elices MJ Hemler ME The human integrin VLA-2 is a collagen receptor on some cells and a collagen/laminin receptor on others. Proc Natl Acad Sci USA (1989) 86:9906–10. 10.1073/pnas.86.24.9906 PMC298611 2557634

[163] Lian XY Zhang W Wu DH Ma JC Zhou JD Zhang ZH Methylation-independent ITGA2 overexpression is associated with poor prognosis in de novo acute myeloid leukemia. J Cell Physiol (2018) 233:9584–93. 10.1002/jcp.26866 30132837

[164] Naci D El Azreq MA Chetoui N Lauden L Sigaux F Charron D alpha2beta1 integrin promotes chemoresistance against doxorubicin in cancer cells through extracellular signal-regulated kinase (ERK). J Biol Chem (2012) 287:17065–76. 10.1074/jbc.M112.349365 PMC3366820 22457358

[165] Naci D Berrazouane S Barabe F Aoudjit F Cell adhesion to collagen promotes leukemia resistance to doxorubicin by reducing DNA damage through the inhibition of Rac1 activation. Sci Rep (2019) 9:19455. 10.1038/s41598-019-55934-w 31857649 PMC6923425

[166] Elices MJ Urry LA Hemler ME Receptor functions for the integrin VLA-3: fibronectin, collagen, and laminin binding are differentially influenced by Arg-Gly-Asp peptide and by divalent cations. J Cell Biol (1991) 112:169–81. 10.1083/jcb.112.1.169 PMC2288801 1986004

[167] Tomellini E Fares I Lehnertz B Chagraoui J Mayotte N MacRae T Integrin-alpha3 Is a Functional Marker of Ex Vivo Expanded Human Long-Term Hematopoietic Stem Cells. Cell Rep (2019) 28:1063–73.e5. 10.1016/j.celrep.2019.06.084 31340144

[168] Yang JT Rayburn H Hynes RO Cell adhesion events mediated by alpha 4 integrins are essential in placental and cardiac development. Development (1995) 121:549–60. 10.1242/dev.121.2.549 7539359

[169] Mahlknecht U Schonbein C Histone deacetylase inhibitor treatment downregulates VLA-4 adhesion in hematopoietic stem cells and acute myeloid leukemia blast cells. Haematologica (2008) 93:443–6. 10.3324/haematol.11796 18268283

[170] Madrazo E Ruano D Abad L Alonso-Gomez E Sanchez-Valdepenas C Gonzalez-Murillo A G9a Correlates with VLA-4 Integrin and Influences the Migration of Childhood Acute Lymphoblastic Leukemia Cells. Cancers (Basel) (2018) 10. 10.3390/cancers10090325 PMC6162492 30213075

[171] Leung KT Zhang C Chan KYY Li K Cheung JTK Ng MHL CD9 blockade suppresses disease progression of high-risk pediatric B-cell precursor acute lymphoblastic leukemia and enhances chemosensitivity. Leukemia (2020) 34:709–20. 10.1038/s41375-019-0593-7 31624373

[172] Bajaj J Konuma T Lytle NK Kwon HY Ablack JN Cantor JM CD98-Mediated Adhesive Signaling Enables the Establishment and Propagation of Acute Myelogenous Leukemia. Cancer Cell (2016) 30:792–805. 10.1016/j.ccell.2016.10.003 27908736 PMC5137811

[173] Layani-Bazar A Skornick I Berrebi A Pauker MH Noy E Silberman A Redox modulation of adjacent thiols in VLA-4 by AS101 converts myeloid leukemia cells from a drug-resistant to drug-sensitive state. Cancer Res (2014) 74:3092–103. 10.1158/0008-5472.CAN-13-2159 24699624

[174] Liu CC Leclair P Yap SQ Lim CJ The membrane-proximal KXGFFKR motif of alpha-integrin mediates chemoresistance. Mol Cell Biol (2013) 33:4334–45. 10.1128/MCB.00580-13 PMC3811891 24001772

[175] Hsieh YT Gang EJ Geng H Park E Huantes S Chudziak D Integrin alpha4 blockade sensitizes drug resistant pre-B acute lymphoblastic leukemia to chemotherapy. Blood (2013) 121:1814–8. 10.1182/blood-2012-01-406272 PMC3591800 23319569

[176] Duchartre Y Bachl S Kim HN Gang EJ Lee S Liu HC Effects of CD49d-targeted antisense-oligonucleotide on alpha4 integrin expression and function of acute lymphoblastic leukemia cells: Results of in vitro and in vivo studies. PLoS One (2017) 12:e0187684. 10.1371/journal.pone.0187684 29117236 PMC5678723

[177] Walter RB Alonzo TA Gerbing RB Ho PA Smith FO Raimondi SC High expression of the very late antigen-4 integrin independently predicts reduced risk of relapse and improved outcome in pediatric acute myeloid leukemia: a report from the children’s oncology group. J Clin Oncol (2010) 28:2831–8. 10.1200/JCO.2009.27.5693 PMC2903318 20421533

[178] Becker PS Kopecky KJ Wilks AN Chien S Harlan JM Willman CL Very late antigen-4 function of myeloblasts correlates with improved overall survival for patients with acute myeloid leukemia. Blood (2009) 113:866–74. 10.1182/blood-2007-12-124818 PMC2630271 18927435

[179] Pytela R Pierschbacher MD Ruoslahti E Identification and isolation of a 140 kd cell surface glycoprotein with properties expected of a fibronectin receptor. Cell (1985) 40:191–8. 10.1016/0092-8674(85)90322-8 3155652

[180] van der Loo JC Xiao X McMillin D Hashino K Kato I Williams DA VLA-5 is expressed by mouse and human long-term repopulating hematopoietic cells and mediates adhesion to extracellular matrix protein fibronectin. J Clin Invest (1998) 102:1051–61. 10.1172/JCI3687 PMC508972 9727075

[181] Joshi I Yoshida T Jena N Qi X Zhang J Van Etten RA Loss of Ikaros DNA-binding function confers integrin-dependent survival on pre-B cells and progression to acute lymphoblastic leukemia. Nat Immunol (2014) 15:294–304. 10.1038/ni.2821 24509510 PMC4494688

[182] Yi L Hu Q Zhou J Liu Z Li H Alternative splicing of Ikaros regulates the FUT4/Le(X)-alpha5beta1 integrin-FAK axis in acute lymphoblastic leukemia. Biochem Biophys Res Commun (2019) 510:128–34. 10.1016/j.bbrc.2019.01.064 30683310

[183] De Toni F Racaud-Sultan C Chicanne G Mas VM Cariven C Mesange F A crosstalk between the Wnt and the adhesion-dependent signaling pathways governs the chemosensitivity of acute myeloid leukemia. Oncogene (2006) 25:3113–22. 10.1038/sj.onc.1209346 16407823

[184] De Toni-Costes F Despeaux M Bertrand J Bourogaa E Ysebaert L Payrastre B A New alpha5beta1 integrin-dependent survival pathway through GSK3beta activation in leukemic cells. PLoS One (2010) 5:e9807. 10.1371/journal.pone.0009807 20352103 PMC2843713

[185] Hu Z Slayton WB Integrin VLA-5 and FAK are Good Targets to Improve Treatment Response in the Philadelphia Chromosome Positive Acute Lymphoblastic Leukemia. Front Oncol (2014) 4:112. 10.3389/fonc.2014.00112 24860788 PMC4030186

[186] Sonnenberg A Modderman PW Hogervorst F Laminin receptor on platelets is the integrin VLA-6. Nature (1988) 336:487–9. 10.1038/336487a0 2973567

[187] Kennel SJ Foote LJ Falcioni R Sonnenberg A Stringer CD Crouse C Analysis of the tumor-associated antigen TSP-180. Identity with alpha 6-beta 4 in the integrin superfamily. J Biol Chem (1989) 264:15515–21. 2475502

[188] Shah Scharff BFS Modvig S Thastrup M Levinsen M Degn M Ryder LP A comprehensive clinical study of integrins in acute lymphoblastic leukemia indicates a role of alpha6/CD49f in persistent minimal residual disease and alpha5 in the colonization of cerebrospinal fluid. Leuk Lymphoma (2020) 61(7):1714–8. 10.1080/10428194.2020.1731500 32107949

[189] Yamakawa N Kaneda K Saito Y Ichihara E Morishita K The increased expression of integrin alpha6 (ITGA6) enhances drug resistance in EVI1(high) leukemia. PLoS One (2012) 7:e30706. 10.1371/journal.pone.0030706 22295105 PMC3266272

[190] Yao H Price TT Cantelli G Ngo B Warner MJ Olivere L Leukaemia hijacks a neural mechanism to invade the central nervous system. Nature (2018) 560:55–60. 10.1038/s41586-018-0342-5 30022166 PMC10257142

[191] Gang EJ Kim HN Hsieh YT Ruan Y Ogana H Pham J Integrin alpha6 mediates drug resistance of acute lymphoblastic B-cell leukemia. Blood (2020) 136(2):210–23. 10.1182/blood.2019001417 PMC7357190 32219444

[192] Liu HC Gang EJ Kim HN Abdel-Azim N Chen R Abdel-Azim H Integrin Antibody Decreases Deformability of Patient-Derived Pre-B Acute Lymphocytic Leukemia Cells as Measured by High-Frequency Acoustic Tweezers. J Ultrasound Med (2020) 39:589–95. 10.1002/jum.15139 PMC7493593 31633840

[193] Yao CC Ziober BL Sutherland AE Mendrick DL Kramer RH Laminins promote the locomotion of skeletal myoblasts via the alpha 7 integrin receptor. J Cell Sci (1996) 109(Pt 13):3139–50. 10.1242/jcs.109.13.3139 9004048

[194] Ziober BL Vu MP Waleh N Crawford J Lin CS Kramer RH Alternative extracellular and cytoplasmic domains of the integrin alpha 7 subunit are differentially expressed during development. J Biol Chem (1993) 268:26773–83. 8253814

[195] Collo G Starr L Quaranta V A new isoform of the laminin receptor integrin alpha 7 beta 1 is developmentally regulated in skeletal muscle. J Biol Chem (1993) 268:19019–24. 8360188

[196] Bao ZZ Lakonishok M Kaufman S Horwitz AF Alpha 7 beta 1 integrin is a component of the myotendinous junction on skeletal muscle. J Cell Sci (1993) 106(Pt 2):579–89. 10.1242/jcs.106.2.579 8282763

[197] Kobayashi N Oda T Takizawa M Ishizaki T Tsukamoto N Yokohama A Integrin alpha7 and Extracellular Matrix Laminin 211 Interaction Promotes Proliferation of Acute Myeloid Leukemia Cells and Is Associated with Granulocytic Sarcoma. Cancers (Basel) (2020) 12. 10.3390/cancers12020363 PMC7072541 32033262

[198] Zeng M Ding S Zhang H Huang Q Ren Y Guo P Predictive value of integrin alpha7 for acute myeloid leukemia risk and its correlation with prognosis in acute myeloid leukemia patients. J Clin Lab Anal (2020) 34:e23151. 10.1002/jcla.23151 31855276 PMC7171313

[199] Palmer EL Ruegg C Ferrando R Pytela R Sheppard D Sequence and tissue distribution of the integrin alpha 9 subunit, a novel partner of beta 1 that is widely distributed in epithelia and muscle. J Cell Biol (1993) 123:1289–97. 10.1083/jcb.123.5.1289 PMC2119880 8245132

[200] Yokosaki Y Palmer EL Prieto AL Crossin KL Bourdon MA Pytela R The integrin alpha 9 beta 1 mediates cell attachment to a non-RGD site in the third fibronectin type III repeat of tenascin. J Biol Chem (1994) 269:26691–6. 7523411

[201] Yokosaki Y Matsuura N Sasaki T Murakami I Schneider H Higashiyama S The integrin alpha(9)beta(1) binds to a novel recognition sequence (SVVYGLR) in the thrombin-cleaved amino-terminal fragment of osteopontin. J Biol Chem (1999) 274:36328–34. 10.1074/jbc.274.51.36328 10593924

[202] Taooka Y Chen J Yednock T Sheppard D The integrin alpha9beta1 mediates adhesion to activated endothelial cells and transendothelial neutrophil migration through interaction with vascular cell adhesion molecule-1. J Cell Biol (1999) 145:413–20. 10.1083/jcb.145.2.413 PMC2133104 10209034

[203] Huang XZ Wu JF Ferrando R Lee JH Wang YL Farese RV Jr. Fatal bilateral chylothorax in mice lacking the integrin alpha9beta1. Mol Cell Biol (2000) 20:5208–15. 10.1128/MCB.20.14.5208-5215.2000 PMC85969 10866676

[204] Cao B Zhang Z Grassinger J Williams B Heazlewood CK Churches QI Therapeutic targeting and rapid mobilization of endosteal HSC using a small molecule integrin antagonist. Nat Commun (2016) 7:11007. 10.1038/ncomms11007 26975966 PMC4796355

[205] Schreiber TD Steinl C Essl M Abele H Geiger K Muller CA The integrin alpha9beta1 on hematopoietic stem and progenitor cells: involvement in cell adhesion, proliferation and differentiation. Haematologica (2009) 94:1493–501. 10.3324/haematol.2009.006072 PMC2770959 19608669

[206] Kavanaugh AF Lightfoot E Lipsky PE Oppenheimer-Marks N Role of CD11/CD18 in adhesion and transendothelial migration of T cells. Analysis utilizing CD18-deficient T cell clones. J Immunol (1991) 146:4149–56. 1710241

[207] Inghirami G Wieczorek R Zhu BY Silber R Dalla-Favera R Knowles DM Differential expression of LFA-1 molecules in non-Hodgkin’s lymphoma and lymphoid leukemia. Blood (1988) 72:1431–4. 10.1182/blood.V72.4.1431.bloodjournal7241431 3048446

[208] Horst E Radaszkiewicz T Hooftman-den Otter A Pieters R van Dongen JJ Meijer CJ Expression of the leucocyte integrin LFA-1 (CD11a/CD18) and its ligand ICAM-1 (CD54) in lymphoid malignancies is related to lineage derivation and stage of differentiation but not to tumor grade. Leukemia (1991) 5:848–53. 1683677

[209] Winter SS Sweatman JJ Lawrence MB Rhoades TH Hart AL Larson RS Enhanced T-lineage acute lymphoblastic leukaemia cell survival on bone marrow stroma requires involvement of LFA-1 and ICAM-1. Br J Haematol (2001) 115:862–71. 10.1046/j.1365-2141.2001.03182.x 11843820

[210] Wright SD Weitz JI Huang AJ Levin SM Silverstein SC Loike JD Complement receptor type three (CD11b/CD18) of human polymorphonuclear leukocytes recognizes fibrinogen. Proc Natl Acad Sci USA (1988) 85:7734–8. 10.1073/pnas.85.20.7734 PMC282267 2971974

[211] Lishko VK Yakubenko VP Ugarova TP Podolnikova NP Leukocyte integrin Mac-1 (CD11b/CD18, alphaMbeta2, CR3) acts as a functional receptor for platelet factor 4. J Biol Chem (2018) 293:6869–82. 10.1074/jbc.RA117.000515 PMC5936813 29540475

[212] Diamond MS Staunton DE de Fougerolles AR Stacker SA Garcia-Aguilar J Hibbs ML ICAM-1 (CD54): a counter-receptor for Mac-1 (CD11b/CD18). J Cell Biol (1990) 111:3129–39. 10.1083/jcb.111.6.3129 PMC2116396 1980124

[213] Graf M Reif S Kroll T Hecht K Nuessler V Schmetzer H Expression of MAC-1 (CD11b) in acute myeloid leukemia (AML) is associated with an unfavorable prognosis. Am J Hematol (2006) 81:227–35. 10.1002/ajh.20526 16550517

[214] Zhang Z Morla AO Vuori K Bauer JS Juliano RL Ruoslahti E The alpha v beta 1 integrin functions as a fibronectin receptor but does not support fibronectin matrix assembly and cell migration on fibronectin. J Cell Biol (1993) 122:235–42. 10.1083/jcb.122.1.235 PMC2119613 8314844

[215] Nishimura SL Sheppard D Pytela R Integrin alpha v beta 8. Interaction with vitronectin and functional divergence of the beta 8 cytoplasmic domain. J Biol Chem (1994) 269:28708–15. 7525578

[216] Pytela R Pierschbacher MD Ruoslahti E A 125/115-kDa cell surface receptor specific for vitronectin interacts with the arginine-glycine-aspartic acid adhesion sequence derived from fibronectin. Proc Natl Acad Sci USA (1985) 82:5766–70. 10.1073/pnas.82.17.5766 PMC390633 2412224

[217] Shah CA Bei L Wang H Altman JK Platanias LC Eklund EA Cooperation between AlphavBeta3 integrin and the fibroblast growth factor receptor enhances proliferation of Hox-overexpressing acute myeloid leukemia cells. Oncotarget (2016) 7:54782–94. 10.18632/oncotarget.10189 PMC5342381 27340869

[218] Kapp TG Rechenmacher F Neubauer S Maltsev OV Cavalcanti-Adam EA Zarka R A Comprehensive Evaluation of the Activity and Selectivity Profile of Ligands for RGD-binding Integrins. Sci Rep (2017) 7:39805. 10.1038/srep39805 28074920 PMC5225454

[219] Sun Q Zhou C Ma R Guo Q Huang H Hao J Prognostic value of increased integrin-beta 1 expression in solid cancers: a meta-analysis. Onco Targets Ther (2018) 11:1787–99. 10.2147/OTT.S155279 PMC5881529 29636624

[220] Berrazouane S Boisvert M Salti S Mourad W Al-Daccak R Barabe F Beta1 integrin blockade overcomes doxorubicin resistance in human T-cell acute lymphoblastic leukemia. Cell Death Dis (2019) 10:357. 10.1038/s41419-019-1593-2 31043590 PMC6494825

[221] El Azreq M-A Naci D Aoudjit F Collagen/β1 integrin signaling up-regulates the ABCC1/MRP-1 transporter in an ERK/MAPK-dependent manner. Mol Biol Cell (2012) 23:3473–84. 10.1091/mbc.e12-02-0132 PMC3431945 22787275

[222] Naci D Berrazouane S Barabé F Aoudjit F Cell adhesion to collagen promotes leukemia resistance to doxorubicin by reducing DNA damage through the inhibition of Rac1 activation. Sci Rep (2019) 9:19455–5. 10.1038/s41598-019-55934-w PMC6923425 31857649

[223] Estrugo D Fischer A Hess F Scherthan H Belka C Cordes N Ligand bound beta1 integrins inhibit procaspase-8 for mediating cell adhesion-mediated drug and radiation resistance in human leukemia cells. PLoS One (2007) 2:e269. 10.1371/journal.pone.0000269 17342203 PMC1800908

[224] van Spriel AB de Keijzer S van der Schaaf A Gartlan KH Sofi M Light A The tetraspanin CD37 orchestrates the alpha(4)beta(1) integrin-Akt signaling axis and supports long-lived plasma cell survival. Sci Signal (2012) 5:ra82. 10.1126/scisignal.2003113 23150881

[225] Kotha J Longhurst C Appling W Jennings LK Tetraspanin CD9 regulates beta 1 integrin activation and enhances cell motility to fibronectin via a PI-3 kinase-dependent pathway. Exp Cell Res (2008) 314:1811–22. 10.1016/j.yexcr.2008.01.024 18358474

[226] Floren M Restrepo Cruz S Termini CM Marjon KD Lidke KA Gillette JM Tetraspanin CD82 drives acute myeloid leukemia chemoresistance by modulating protein kinase C alpha and β1 integrin activation. Oncogene (2020) 39:3910–25. 10.1038/s41388-020-1261-0 PMC7210072 32203165

[227] Bui T Rennhack J Mok S Ling C Perez M Roccamo J Functional Redundancy between beta1 and beta3 Integrin in Activating the IR/Akt/mTORC1 Signaling Axis to Promote ErbB2-Driven Breast Cancer. Cell Rep (2019) 29:589–602 e6. 10.1016/j.celrep.2019.09.004 31618629

[228] Miller PG Al-Shahrour F Hartwell KA Chu LP Jaras M Puram RV In Vivo RNAi screening identifies a leukemia-specific dependence on integrin beta 3 signaling. Cancer Cell (2013) 24:45–58. 10.1016/j.ccr.2013.05.004 23770013 PMC3746037

[229] Zhang PF Li KS Shen YH Gao PT Dong ZR Cai JB Galectin-1 induces hepatocellular carcinoma EMT and sorafenib resistance by activating FAK/PI3K/AKT signaling. Cell Death Dis (2016) 7:e2201. 10.1038/cddis.2015.324 27100895 PMC4855644

[230] Su X Esser AK Amend SR Xiang J Xu Y Ross MH Antagonizing Integrin beta3 Increases Immunosuppression in Cancer. Cancer Res (2016) 76:3484–95. 10.1158/0008-5472.CAN-15-2663 PMC4944657 27216180

[231] Postigo AA Sanchez-Mateos P Lazarovits AI Sanchez-Madrid F de Landazuri MO Alpha 4 beta 7 integrin mediates B cell binding to fibronectin and vascular cell adhesion molecule-1. Expression and function of alpha 4 integrins on human B lymphocytes. J Immunol (1993) 151:2471–83. 7689608

[232] Tidswell M Pachynski R Wu SW Qiu SQ Dunham E Cochran N Structure-function analysis of the integrin beta 7 subunit: identification of domains involved in adhesion to MAdCAM-1. J Immunol (1997) 159:1497–505. 9233649

[233] Katayama Y Hidalgo A Peired A Frenette PS Integrin alpha4beta7 and its counterreceptor MAdCAM-1 contribute to hematopoietic progenitor recruitment into bone marrow following transplantation. Blood (2004) 104:2020–6. 10.1182/blood-2003-12-4157 15161666

[234] Dolcetti R Giardini R Doglioni C Cariati R Pomponi F D’Orazi C Alpha 4 beta 7 integrin expression is associated with the leukemic evolution of human and murine T-cell lymphoblastic lymphomas. Am J Pathol (1997) 150:1595–605. PMC1858193 9137086

[235] Chen H Hori T Maeda M Uchiyama T Identification of an adhesion molecule expressed on adult T cell leukemia cells derived from a patient with gastrointestinal involvement: implication for a possible role of integrin beta 7 in leukemic cell infiltration into intestinal mucosa. J Clin Immunol (1999) 19:186–93. 10.1023/a:1020507828066 10404404

[236] Nigim F Kiyokawa J Gurtner A Kawamura Y Hua L Kasper EM A Monoclonal Antibody Against beta1 Integrin Inhibits Proliferation and Increases Survival in an Orthotopic Model of High-Grade Meningioma. Target Oncol (2019) 14:479–89. 10.1007/s11523-019-00654-4 31301014

[237] Hosen N Matsunaga Y Hasegawa K Matsuno H Nakamura Y Makita M The activated conformation of integrin beta7 is a novel multiple myeloma-specific target for CAR T cell therapy. Nat Med (2017) 23:1436–43. 10.1038/nm.4431 29106400

[238] Wallstabe L Mades A Frenz S Einsele H Rader C Hudecek M CAR T cells targeting alphavbeta3 integrin are effective against advanced cancer in preclinical models. Adv Cell Gene Ther (2018) 1. 10.1002/acg2.11 PMC6226260 30420973

[239] Whilding LM Parente-Pereira AC Zabinski T Davies DM Petrovic RMG Kao YV Targeting of Aberrant alphavbeta6 Integrin Expression in Solid Tumors Using Chimeric Antigen Receptor-Engineered T Cells. Mol Ther (2017) 25:259–73. 10.1016/j.ymthe.2016.10.012 PMC5261028 28129120

[240] Mateo J Berlin J de Bono JS Cohen RB Keedy V Mugundu G A first-in-human study of the anti-alpha5beta1 integrin monoclonal antibody PF-04605412 administered intravenously to patients with advanced solid tumors. Cancer Chemother Pharmacol (2014) 74:1039–46. 10.1007/s00280-014-2576-8 PMC4209234 25212537

[241] Alday-Parejo B Stupp R Ruegg C Are Integrins Still Practicable Targets for Anti-Cancer Therapy? Cancers (Basel) (2019) 11. 10.3390/cancers11070978 PMC6678560 31336983

[242] Colombel JF Sands BE Rutgeerts P Sandborn W Danese S D’Haens G The safety of vedolizumab for ulcerative colitis and Crohn’s disease. Gut (2017) 66:839–51. 10.1136/gutjnl-2015-311079 PMC5531223 26893500

[243] Noman M Ferrante M Bisschops R De Hertogh G Van den Broeck K Rans K Vedolizumab Induces Long-term Mucosal Healing in Patients With Crohn’s Disease and Ulcerative Colitis. J Crohns Colitis (2017) 11:1085–9. 10.1093/ecco-jcc/jjx048 28369329

[244] Ng CM Bai S Takimoto CH Tang MT Tolcher AW Mechanism-based receptor-binding model to describe the pharmacokinetic and pharmacodynamic of an anti-alpha5beta1 integrin monoclonal antibody (volociximab) in cancer patients. Cancer Chemother Pharmacol (2010) 65:207–17. 10.1007/s00280-009-1023-8 19468731

[245] Ramakrishnan V Bhaskar V Law DA Wong MH DuBridge RB Breinberg D Preclinical evaluation of an anti-alpha5beta1 integrin antibody as a novel anti-angiogenic agent. J Exp Ther Oncol (2006) 5:273–86. 17024968

[246] Ricart AD Tolcher AW Liu G Holen K Schwartz G Albertini M Volociximab, a chimeric monoclonal antibody that specifically binds alpha5beta1 integrin: a phase I, pharmacokinetic, and biological correlative study. Clin Cancer Res (2008) 14:7924–9. 10.1158/1078-0432.CCR-08-0378 PMC3394092 19047123

[247] Bell-McGuinn KM Matthews CM Ho SN Barve M Gilbert L Penson RT single-arm study of the anti-alpha5beta1 integrin antibody volociximab as monotherapy in patients with platinum-resistant advanced epithelial ovarian or primary peritoneal cancer. Gynecol Oncol (2011) 121:273–9. 10.1016/j.ygyno.2010.12.362 PMC4426879 21276608

[248] Cianfrocca ME Kimmel KA Gallo J Cardoso T Brown MM Hudes G Phase 1 trial of the antiangiogenic peptide ATN-161 (Ac-PHSCN-NH(2)), a beta integrin antagonist, in patients with solid tumours. Br J Cancer (2006) 94:1621–6. 10.1038/sj.bjc.6603171 PMC2361324 16705310

[249] Sui A Zhong Y Demetriades AM Shen J Su T Yao Y ATN-161 as an Integrin alpha5beta1 Antagonist Depresses Ocular Neovascularization by Promoting New Vascular Endothelial Cell Apoptosis. Med Sci Monit (2018) 24:5860–73. 10.12659/MSM.907446 PMC6116638 30133427

[250] Stoeltzing O Liu W Reinmuth N Fan F Parry GC Parikh AA Inhibition of integrin alpha5beta1 function with a small peptide (ATN-161) plus continuous 5-FU infusion reduces colorectal liver metastases and improves survival in mice. Int J Cancer (2003) 104:496–503. 10.1002/ijc.10958 12584749

[251] Chen Q Manning CD Millar H McCabe FL Ferrante C Sharp C CNTO 95, a fully human anti alphav integrin antibody, inhibits cell signaling, migration, invasion, and spontaneous metastasis of human breast cancer cells. Clin Exp Metastasis (2008) 25:139–48. 10.1007/s10585-007-9132-4 18064530

[252] Moschos SJ Sander CA Wang W Reppert SL Drogowski LM Jukic DM Pharmacodynamic (phase 0) study using etaracizumab in advanced melanoma. J Immunother (2010) 33:316–25. 10.1097/CJI.0b013e3181c1f216 20445352

[253] McNeel DG Eickhoff J Lee FT King DM Alberti D Thomas JP Phase I trial of a monoclonal antibody specific for alphavbeta3 integrin (MEDI-522) in patients with advanced malignancies, including an assessment of effect on tumor perfusion. Clin Cancer Res (2005) 11:7851–60. 10.1158/1078-0432.CCR-05-0262 16278408

[254] Jiang Y Dai J Yao Z Shelley G Keller ET Abituzumab Targeting of alphaV-Class Integrins Inhibits Prostate Cancer Progression. Mol Cancer Res (2017) 15:875–83. 10.1158/1541-7786.MCR-16-0447 PMC5541673 28314844

[255] Hussain M Le Moulec S Gimmi C Bruns R Straub J Miller K Differential Effect on Bone Lesions of Targeting Integrins: Randomized Phase II Trial of Abituzumab in Patients with Metastatic Castration-Resistant Prostate Cancer. Clin Cancer Res (2016) 22:3192–200. 10.1158/1078-0432.CCR-15-2512 26839144

[256] Haddad T Qin R Lupu R Satele D Eadens M Goetz MP A phase I study of cilengitide and paclitaxel in patients with advanced solid tumors. Cancer Chemother Pharmacol (2017) 79:1221–7. 10.1007/s00280-017-3322-9 PMC9594487 28477227

[257] Zhang L Gulses A Purcz N Weimer J Wiltfang J Acil Y A comparative assessment of the effects of integrin inhibitor cilengitide on primary culture of head and neck squamous cell carcinoma (HNSCC) and HNSCC cell lines. Clin Transl Oncol (2019) 21:1052–60. 10.1007/s12094-018-02025-3 30632010

[258] Nabors LB Fink KL Mikkelsen T Grujicic D Tarnawski R Nam DH Two cilengitide regimens in combination with standard treatment for patients with newly diagnosed glioblastoma and unmethylated MGMT gene promoter: results of the open-label, controlled, randomized phase II CORE study. Neuro Oncol (2015) 17:708–17. 10.1093/neuonc/nou356 PMC4482861 25762461

[259] Weller M Nabors LB Gorlia T Leske H Rushing E Bady P Cilengitide in newly diagnosed glioblastoma: biomarker expression and outcome. Oncotarget (2016) 7:15018–32. 10.18632/oncotarget.7588 PMC4924768 26918452

[260] Cirkel GA Kerklaan BM Vanhoutte F Van der Aa A Lorenzon G Namour F A dose escalating phase I study of GLPG0187, a broad spectrum integrin receptor antagonist, in adult patients with progressive high-grade glioma and other advanced solid malignancies. Invest New Drugs (2016) 34:184–92. 10.1007/s10637-015-0320-9 PMC4786599 26792581

[261] Reeves KJ Hurrell JE Cecchini M van der Pluijm G Down JM Eaton CL Prostate cancer cells home to bone using a novel in vivo model: modulation by the integrin antagonist GLPG0187. Int J Cancer (2015) 136:1731–40. 10.1002/ijc.29165 25156971

[262] van der Horst G van den Hoogen C Buijs JT Cheung H Bloys H Pelger RC Targeting of alpha(v)-integrins in stem/progenitor cells and supportive microenvironment impairs bone metastasis in human prostate cancer. Neoplasia (2011) 13:516–25. 10.1593/neo.11122 PMC3114245 21677875

[263] Carbonell WS DeLay M Jahangiri A Park CC Aghi MK beta1 integrin targeting potentiates antiangiogenic therapy and inhibits the growth of bevacizumab-resistant glioblastoma. Cancer Res (2013) 73:3145–54. 10.1158/0008-5472.CAN-13-0011 PMC4040366 23644530

